# Yeast histone H3 lysine 4 demethylase Jhd2 regulates mitotic ribosomal DNA condensation

**DOI:** 10.1186/s12915-014-0075-3

**Published:** 2014-09-24

**Authors:** Hong-Yeoul Ryu, Seong Hoon Ahn

**Affiliations:** Division of Molecular and Life Sciences, College of Science and Technology, Hanyang University, 1271 Sa 3-dong, Sangnok-gu, Ansan, Gyeonggi-do 426-791 Republic of Korea

**Keywords:** Jhd2, histone demethylation, RENT, condensin, rDNA silencing

## Abstract

**Background:**

Nucleolar ribosomal DNA is tightly associated with silent heterochromatin, which is important for rDNA stability, nucleolar integration and cellular senescence. Two pathways have been described that lead to rDNA silencing in yeast: 1) the RENT (regulator of nucleolar silencing and telophase exit) complex, which is composed of Net1, Sir2 and Cdc14 and is required for Sir2-dependent rDNA silencing; and 2) the Sir2-independent silencing mechanism, which involves the Tof2 and Tof2-copurified complex, made up of Lrs4 and Csm1. Here, we present evidence that changes in histone H3 lysine methylation levels distinctly regulate rDNA silencing by recruiting different silencing proteins to rDNA, thereby contributing to rDNA silencing and nucleolar organization in yeast.

**Results:**

We found that Lys4, Lys79 and Lys36 methylation within histone H3 acts as a bivalent marker for the regulation of rDNA recombination and RENT complex-mediated rDNA silencing, both of which are Sir2-dependent pathways. By contrast, we found that Jhd2, an evolutionarily conserved JARID1 family H3 Lys4 demethylase, affects all states of methylated H3K4 within the nontranscribed spacer (NTS) regions of rDNA and that its activity is required for the regulation of rDNA silencing in a Sir2-independent manner. In this context, Jhd2 regulates rDNA recombination through the Tof2/Csm1/Lrs4 pathway and prevents excessive recruitment of Tof2, Csm1/Lrs4 and condensin subunits to the replication fork barrier site within the NTS1 region. Our FISH analyses further demonstrate that the demethylase activity of Jhd2 regulates mitotic rDNA condensation and that *JHD2*-deficient cells contain the mostly hypercondensed rDNA mislocalized away from the nuclear periphery.

**Conclusions:**

Our results show that yeast Jhd2, which demethylates histone H3 Lys4 near the rDNA locus, regulates rDNA repeat stability and rDNA silencing in a Sir2-independent manner by maintaining Csm1/Lrs4 and condensin association with rDNA regions during mitosis. These data suggest that Jhd2-mediated alleviation of excessive Csm1/Lrs4 or condensin at the NTS1 region of rDNA is required for the integrity of rDNA repeats and proper rDNA silencing during mitosis.

**Electronic supplementary material:**

The online version of this article (doi:10.1186/s12915-014-0075-3) contains supplementary material, which is available to authorized users.

## Background

Histones have N- and C-terminal tails and core fold domains that are subject to multiple post-translational modifications such as acetylation, phosphorylation, ubiquitylation and methylation. The lysine residues of histones can be mono-, di- or trimethylated (Kme1, Kme2 and Kme3) and serve as docking sites for various regulatory factors including transcription factors, chromatin-remodeling complexes or histone-modifying enzymes, depending on which lysine residue is methylated. In general, histone H3 methylation at K4 (H3K4), K36 (H3K36) or K79 (H3K79) is primarily associated with gene activation, whereas H3 methylation at K9 or K27 and H4 methylation at K20 are mainly associated with gene repression (reviewed in [1]).

It is now evident that histone methylation is dynamically regulated by histone demethylases. In the yeast *Saccharomyces cerevisiae*, there are five Jumonji C (JmjC) domain-containing histone demethylases (JHDMs): Jhd1, Jhd2, Rph1, Gis1 and Ecm5. Jhd1/Kdm2 is a JHDM1 ortholog and has demethylase activity toward H3K36me1 and H3K36me2 *in vitro* and *in vivo* [2,3]. This demethylase is necessary for promoting transcription elongation through the removal of H3K36 methylation, a repressive chromatin mark generated by Set2 [4]. Jhd2/Kdm5 is an evolutionarily conserved JARID1 family protein that has demethylase activity toward H3K4me3 *in vitro* or *in vivo* [3,5]. Other groups have also shown that Jhd2 demethylates all forms of H3K4 methylation *in vitro* or *in vivo* [6,7]. Demethylation by Jhd2 was originally shown to regulate telomeric silencing; loss of Jhd2 enhances telomeric silencing, and overexpression of *JHD2* counteracts this effect [5]. Additionally, the ability of Jhd2 to associate with chromatin to modulate H3K4 methylation on both active and repressed genes has been reported [6]. The Rph1/Kdm4 and Gis1 demethylases are JHDM3/JMJD2 orthologs and have *in vivo* demethylase activity toward H3K36me3/2 and H3K36me2/1, respectively [3]. Although Rph1, like Jhd1, positively affects transcription [4], little is known about Gis1. Ecm5 contains a JmjC domain, but its demethylase activity is still unclear [3].

In eukaryotic genomes, rRNA genes (rDNA) exist in multicopy tandem arrays with a variable number of units among organisms, ranging from fewer than 100 to more than 10,000 copies. In humans, the clusters of rDNA repeats, termed nucleolar organizer regions (NORs), are composed of 300 to 400 copies per haploid genome of a 43-kb unit and are located on five different chromosomes: 13, 14, 15, 21 and 22 [8]. The budding yeast rDNA is organized with 100 to 200 copies of tandemly repeated 9.1-kb units on chromosome XII and is localized to the nucleolus. This rDNA unit consists of a 5S rRNA gene that is transcribed by RNA polymerase III and a 35S pre-rRNA gene that is transcribed by RNA polymerase I; the two genes are separated by two nontranscribed spacers (NTSs), NTS1 and NTS2 [9]. The nucleolar rDNA is tightly associated with silent heterochromatin, which serves an important role in nucleolar function. Indeed, loss of silencing in the rDNA regions correlates with rDNA instability, nucleolar disintegration and cellular senescence [8]. The RENT complex, which is composed of Net1, Sir2 and Cdc14, is required for rDNA silencing. Net1 is the core subunit of the RENT complex, and it tethers Sir2 to rDNA, regulating the exit from mitosis by sequestering Cdc14 until the telophase [10–12]. In particular, Net1 and Sir2 are preferentially associated with both the NTS1 and NTS2/Polymerase I promoter regions. Fob1 is required for rDNA silencing through the recruitment of Net1 and Sir2 to the replication fork barrier (RFB) site within NTS1, a region that overlaps the E element of the mitotic recombination hotspot *HOT1* [13]. Little is known about how Net1 or Sir2 associate with NTS2.

Recent studies suggest a distinct mechanism that regulates rDNA silencing, separate from the Sir2-dependent pathway. This Sir2-independent silencing mechanism includes Tof2 and two additional proteins, Csm1 and Lrs4, which are subunits of the previously identified monopolin complex that is required for co-orientation in meiosis I [14]. Tof2 is required for rDNA silencing and is primarily recruited to NTS1 through association with Fob1 [14,15]. Likewise, both Csm1 and Lrs4 primarily associate with NTS1 in a Fob1- and Tof2-dependent manner and are required for silencing at NTS1 as well. Indeed, the deletion of either *CSM1* or *LRS4* has little effect on Sir2 localization to NTS1, and vice versa [14]. Thus, so far, two pathways have been described that lead to rDNA silencing in yeast: a Sir2-dependent pathway, in which Fob1 recruits Net1, leading to Sir2 association with NTS1, and a Sir2-independent pathway, in which Fob1 alternatively recruits Tof2 to NTS1, leading to Csm1/Lrs4 recruitment.

In the present study, we explored the role of JmjC-containing demethylases in regulating heterochromatin silencing. Our results show that yeast Jhd2 demethylates histone H3K4 near the rDNA locus and that this activity is required for Sir2-independent rDNA silencing. We suggest that Jhd2 is the exclusive demethylase that regulates nucleolar organization by maintaining the association of condensin with the RFB site within the NTS1 region during the mitotic phase of the cell cycle.

## Results

### Five yeast JmjC demethylases contribute to telomeric silencing

**Figure 1:**
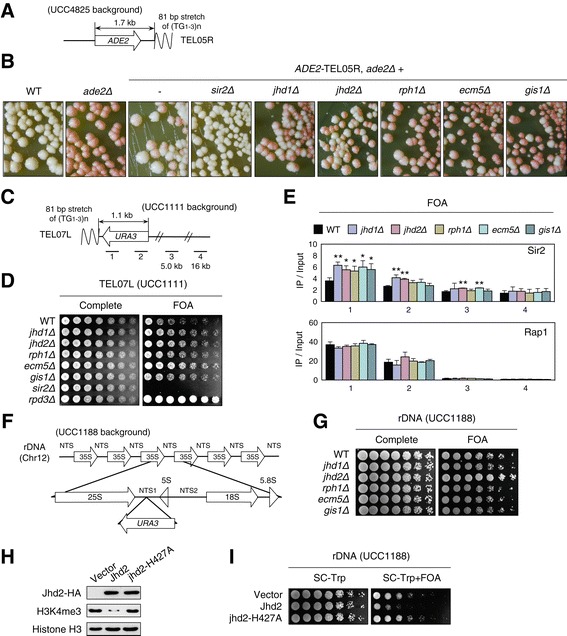
**The demethylase activity of Jhd2 is required for the regulation of rDNA silencing. (A)** Schematic diagram of the *ADE2* reporter integrated at TEL05R. **(B)** *ADE2*-based telomeric silencing assays were performed for the indicated strains. *ADE2-TEL05R* strains contain an *ADE2* reporter as shown in **(A)**. **(C)** Schematic representation of the *URA3* reporter integrated at TEL07L. The bars and numbers below the subtelomeric regions indicate the relative positions of the ChIP PCR products in **(E)** and the distances from telomeric DNA sequences (TG_1–3_). **(D)** *URA3*-based telomeric silencing assays for the indicated strains. **(E)** ChIP analyses were performed using antibodies against Sir2 or Rap1 for the indicated strains with a *URA3* reporter. Cells grown to an A_600_ of 0.1 in synthetic complete (SC) medium were transferred to SC medium containing 0.1% 5-fluoroorotic acid (FOA). Error bars indicate the standard deviation (SD) calculated from PCRs performed using three independent chromatin preparations. Asterisks indicate statistically significant differences determined by pairwise comparisons between wild type (WT) and each of the indicated mutants using a two-tailed Student’s *t* test (*, *P* <0.05; **, *P* <0.01). **(F)** Schematic of the *URA3* reporter integrated at the NTS1 region of rDNA. **(G, I)** *URA3*-based rDNA silencing assays for the indicated strains. For the silencing assay in **(I)**, strains containing empty vector (pRS424), constructs expressing hemagglutinin (HA)-tagged WT Jhd2, or the H427A mutant were grown in SC medium lacking Trp and then serially diluted and spotted onto SC-Trp or SC-Trp medium containing 0.1% FOA. **(H)** Strains used in **(I)** were grown in SC medium lacking Trp and histone H3K4me3 levels were analyzed using immunoblotting assays with an anti-H3K4me3 antibody. As controls, anti-HA and anti-H3 antibodies were used to determine the protein levels of Jhd2 and histone H3, respectively. bp, base pair; ChIP, chromatin immunoprecipitation; Chr, chromosome; FOA, 5-fluoroorotic acid; IP, immunoprecipitation; kb, kilobase; SC, synthetic complete; WT, wild type.

To investigate whether most JmjC-containing demethylases play a similar role in telomeric silencing to that of Jhd2, we performed silencing assays using an *ADE2* reporter gene integrated at telomere-proximal regions on the right arm of telomere V (TEL05R) (Figure 1A). Normally, cells expressing a wild-type (WT) copy of *ADE2* produce white colonies, whereas those deficient in this gene appear red due to the accumulation of red pigment [16]. However, when the *ADE2* gene is placed at a telomere, its transcriptional state stochastically switches, which is evident by the presence of individual colonies composed of red and white sectors [17]. Consistently, we observed that the *ADE2* reporter produced colonies composed of red and white sectors, whereas the loss of Sir2 resulted in mostly white colonies. Interestingly, we found that deletion mutants for each JmjC demethylase produced colonies with larger red sectors, indicating that the role of yeast JmjC-demethylases in regulating telomeric silencing is not restricted to Jhd2 (Figure 1B).

To ascertain the common role of yeast JmjC demethylases in regulating telomeric silencing, we performed silencing assays in cells containing a *URA3*-reporter gene embedded in the telomere-proximal regions on the left arm of telomere VII (TEL07L) (Figure 1C). The drug 5-fluoroorotic acid (FOA), which is converted into a lethal metabolite by active *URA3* enzyme, was added to cells to evaluate the level of silencing. As the results for TEL05R, we observed that telomeric silencing was enhanced in all deletion mutants for each JmjC-demethylase at TEL07L (Figure 1D). As previously reported, cells lacking Sir2 or Rpd3 repressed or enhanced telomeric silencing, respectively [18]. Consistent with this, chromatin immunoprecipitation (ChIP) analyses showed that the association of Sir2 with telomere-proximal regions was significantly increased by the loss of each demethylase, whereas Rap1 association was unaffected (Figure 1E). Consequently, our results show that the five JmjC-containing demethylases in yeast share common functions in the regulation of telomeric silencing.

### Jhd2 exclusively contributes to rDNA silencing

In the yeast *S. cerevisiae*, heterochromatic-like regions under the control of Sir2 include telomeric regions, rDNA and silenced mating-type loci [19]. The initial observation that telomeric silencing was enhanced in cells deficient for any JmjC-demethylase led us to examine whether silencing at rDNA regions and mating-type loci was affected by the loss of demethylases. To test this, we used a *URA3* reporter integrated into the NTS1 region of the rDNA (Figure 1F). In striking contrast with the common functions of yeast JmjC demethylases in telomeric silencing, we found that only *jhd2Δ* profoundly enhanced rDNA silencing; the addition of FOA potentiated cell growth (Figure 1G).

To examine further whether the effect of Jhd2 on rDNA silencing was dependent on the enzymatic activity of Jhd2, we used a catalytically inactive mutant of Jhd2 (jhd2-H427A), which contains a mutation in the Fe(II) binding site. As reported previously, we observed that overexpression of WT Jhd2 significantly reduced H3K4me3 levels, whereas overexpression of jhd2-H427A had little effect on H3K4me3 (Figure 1H) [5,7]. Moreover, rDNA silencing was significantly reduced in Jhd2-overexpressing cells, but not in jhd2-H427A-overexpressing cells (Figure 1I), indicating that the regulation of rDNA silencing is completely dependent on the demethylase activity of Jhd2. By contrast, the growth of all JmjC-demethylase deletion mutants – which contained *URA3* reporters integrated into the *HMR* or *HML* loci – was indistinguishable from that of an isogenic WT strain (Additional file 1: Figure S1). In cells lacking Sir2 or Rpd3, we observed repression and enhancement of silencing, respectively, at *HM* loci, as previously reported [18,20]. Taken together, our data reveal that Jhd2 is the only JmjC demethylase that contributes to the regulation of rDNA silencing.

### Bivalent regulation of rDNA silencing by H3 lysine methylases

The observation that loss of Jhd2 exclusively enhanced rDNA silencing raises several questions. If, among the five JmjC demethylases, Jhd2 is solely responsible for rDNA silencing, is the association of silencing proteins with rDNA affected only by the loss of Jhd2? Jhd2 is a demethylase specific for histone H3K4. Thus, does Set1, the methylase for histone H3K4, have the opposite effect on rDNA silencing? If so, does Set1 regulate silencing through the same pathway as Jhd2?

**Figure 2:**
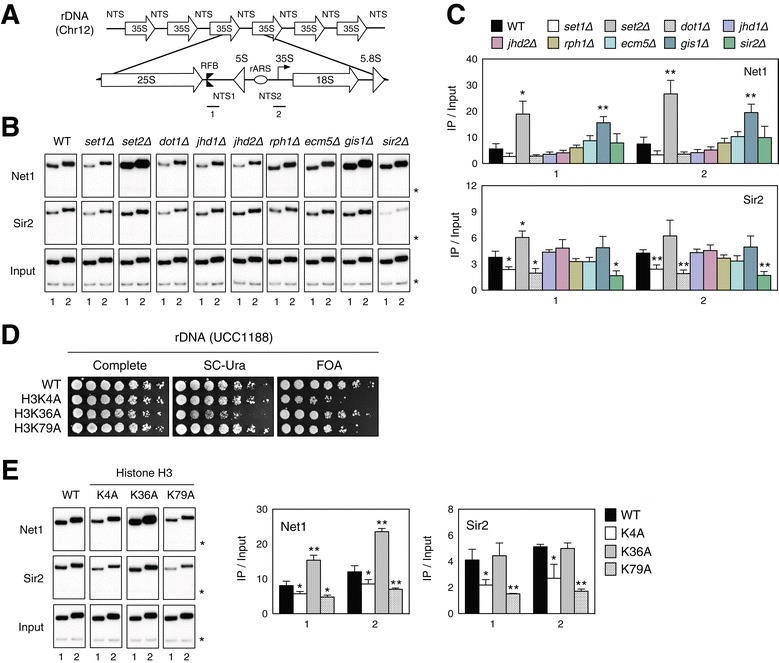
**Changes in RENT component recruitment at NTS regions due to loss of H3 methylases or demethylases. (A)** Schematic diagram of an rDNA unit embedded within a tandem array on chromosome XII. The 35S pre-rRNA encoding the 18S, 5.8S and 25S rRNAs is separated by NTS1 and NTS2. The locations of RFB (double triangle), replication origin ARS (oval), 5S rRNA gene (triangle), and 35S transcription start site (bent arrow) are shown. The bars and numbers below the NTS regions indicate the positions of the ChIP PCR products in **(B)** and **(E)** and those used in all subsequent ChIP experiments. **(B)** The association of Net1 and Sir2 with rDNA regions was analyzed by ChIP using immunoglobulin G (IgG)-Sepharose or a Sir2 antibody in WT or the indicated deletion strains carrying TAP-tagged *NET1*. The upper bands in each pane indicate PCR products amplified by the primer sets shown in **(A)** and the lower bands marked by the asterisks are internal controls amplified from untranscribed regions on chromosome V. The bottom panels show PCR products from the input DNA. **(C)** Quantitation of the ChIP results in **(B)**. Error bars indicate the SD from three PCRs performed using two independent chromatin preparations, and asterisks indicate statistically significant differences compared with WT (*, *P* <0.05; **, *P* <0.01). **(D)** *URA3*-based silencing assays at the rDNA region were performed in WT and H3K4A, H3K36A and H3K79A mutant strains. **(E)** The association of Net1 and Sir2 with the rDNA regions was analyzed in WT and H3K4A, H3K36A and H3K79A mutant cells as shown in **(B)**. Each quantified result is shown on the right. Error bars show the SD from three PCRs with two independent chromatin preparations, and asterisks indicate statistically significant differences compared with WT (*, *P* <0.05; **, *P* <0.01). Chr, chromosome; FOA, 5-fluoroorotic acid; IgG, immunoglobulin G; IP, immunoprecipitation; WT, wild type; ARS, autonomously replicating sequence.

To address the first question, we examined the recruitment of the RENT components Net1 and Sir2 to the NTS1 and NTS2 sites of rDNA using ChIP. The NTS1 primer set spans the RFB site, and the NTS2 primer set overlaps the polymerase I transcription initiation region for the 35S rRNA gene (Figure 2A). Consistent with a previous report, we observed that PCR with these two primer pairs amplified two preferential localization sites for both Net1 and Sir2 within the NTS1 and NTS2 regions (Figure 2B,C) [13]. In addition, we confirmed that loss of Sir2 did not affect the association of Net1 with either the NTS1 or NTS2 region, as previously reported [12]. Surprisingly, we found that the association of neither Net1 nor Sir2 with the NTS regions was affected by loss of Jhd2. In addition, we unexpectedly observed that the association of Net1, but not Sir2, was increased up to twofold in *gis1Δ* cells. In silencing assays, the loss of Gis1 had little effect on rDNA silencing (Figure 1G). These data strongly suggest that Jhd2 functions in rDNA silencing through a pathway that is independent of Net1 and Sir2.

Previously, our group and others have reported that the deletion of *SET1* impairs rDNA silencing [20,21]. This observation prompted us to determine whether Set1-mediated H3K4 methylation regulates the association of Net1 or Sir2 with the rDNA locus. As expected, rDNA silencing was impaired by an H3K4A substitution mutation (Figure 2D). Consistent with this finding, the recruitment of both Net1 and Sir2 to NTS1 and NTS2 was compromised in *set1Δ* cells (Figure 2B,C). A *SET1* deletion was previously shown to impair rDNA silencing, but this mutation caused no detectable defect in Net1 and Sir2 recruitment to rDNA [22]. However, our data show that the deletion of *SET1* did reduce the recruitment of Net1 and Sir2 (Figure 2B,C). ChIP analyses for H3K4A mutant cells further support the conclusion that H3K4 methylation is required for the association of both Net1 and Sir2 (Figure 2E). Similarly, defects in rDNA silencing and in the association of Net1 and Sir2 were observed for H3K79A mutant cells, as previously reported (Figure 2D,E) [23]. In parallel, Net1 and Sir2 recruitment was decreased in *dot1Δ* cells (Figure 2B,C)*.* Recently, it has been suggested that Set2, an H3K36 methylase, has an antagonistic role within heterochromatic regions; mutations in H3K36 or *set2∆* cause ectopic silencing adjacent to *HMRa* [24], and the loss of Set2 leads to increased silencing at telomeric, rDNA and *HMR* regions [20]. Consistent with these observations, *set2Δ* significantly increased Net1 and Sir2 recruitment to rDNA (Figure 2B,C). For H3K36A mutant cells, we confirmed enhanced rDNA silencing and significantly increased Net1 recruitment, but were unable to find any significant changes in Sir2 levels (Figure 2D,E). Taken together, these results elucidate the different roles of histone H3 methylases in regulating Net1/Sir2 recruitment to rDNA regions and the resultant rDNA silencing. In particular, both H3K4 and H3K79 methylation by Set1 and Dot1 positively regulate rDNA silencing, whereas H3K36 methylation by Set2 has the opposite effect.

### Jhd2 and Gis1 demethylate H3K4 and H3K36, respectively, within rDNA regions *in vivo*

**Figure 3:**
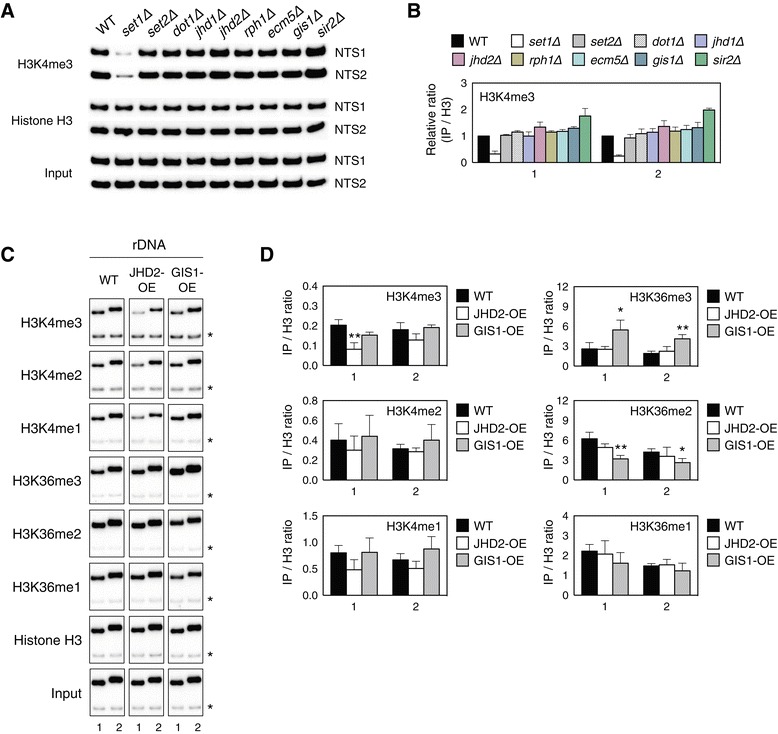
**Jhd2 and Gis1 demethylate H3K4 and H3K36, respectively, within the rDNA regions** ***in vivo.*** **(A)** The levels of histone H3K4 methylation were analyzed using ChIP for the indicated strains. Antibodies against H3K4me3 or H3 were used. **(B)** Quantitation of the ChIP results in **(A)**. The results for methyl-H3 were normalized to the total H3 signal and presented as fold enrichment relative to WT. Error bars indicate the SD from three PCRs performed using two independent chromatin preparations. **(C)** The levels of H3K4 and H3K36 methylation were analyzed by ChIP as shown in Figure 2B. Chromatin fractions were obtained from WT (BY4741) strains containing pRS325-GALpro, pRS325-GALpro-Jhd2-HA or pRS325-GALpro-Gis1-HA. Cells were grown on SC medium containing 2% galactose, followed by immunoprecipitation with the indicated antibodies. **(D)** Quantitation of the ChIP results shown in **(C)**. The results were normalized to an internal control as shown in Figure 2C and to the total H3 signal. Error bars indicate the SD from three PCRs performed using two independent chromatin preparations, and asterisks indicate statistically significant differences compared with WT (*, *P* <0.05; **, *P* <0.01). IP, immunoprecipitation; WT, wild type.

Our genetic and biochemical analyses of each gene deletion and each substitution mutant demonstrate the bivalent regulation of rDNA silencing by the methylases Set1, Dot1 and Set2 through a Sir2-dependent pathway. However, the observation that Jhd2 demethylase regulates rDNA silencing independently of Net1 and Sir2 raises the question of whether Jhd2 demethylase activity is present within the rDNA region. In addition, it remains to be determined whether the loss of Gis1 demethylase activity leads to enhanced Net1 recruitment to the rDNA locus in *gis1Δ* cells. It has been argued that *gis1Δ* decreases the global levels of H3K36me3 but increases the levels of H3K36me2/1 *in vivo* [3]. Consistently, we found that cells lacking Gis1 exhibited reduced levels of H3K36me3 and increased levels of H3K36me2/1 at both NTS1 and NTS2 within the rDNA locus *in vivo* (Additional file 1: Figure S2). As a control, we confirmed that loss of Set2 depleted all three states of H3K36 methylation in this region. Moreover, overexpression of Gis1 decreased both H3K36me1 and H3K36me2, but it increased H3K36me3 (Figure 3C,D). As such, these results indicate that Gis1 affects H3K36me2/1 at the rDNA locus.

Conventionally, H3K4me3 is highly correlated with transcriptional activity, as it is most concentrated at the promoters and 5′ ends of the coding regions of active genes [25]. At the actively transcribed *PMA1* gene, the deletion or overexpression of *JHD2* leads to a twofold increase or decrease, respectively, in H3K4me3 at the promoter or ORF regions of the gene [6]. However, it is unknown whether Jhd2 modulates H3K4 methylation at the rDNA region, although it has been observed that H3K4 methylation can be abrogated by a *set1Δ* or H3K4R substitution mutation at this region [21]. Our ChIP analyses involving α-H3K4me3 also showed that H3K4me3 was repressed by *set1Δ* and slightly increased by *jhd2Δ* at both NTS regions (Figure 3A,B). Furthermore, *JHD2* overexpression caused a decrease in H3K4me3 at both NTS regions, with a significantly stronger reduction at NTS1 compared with NTS2. A considerable decrease in H3K4me2/1 upon *JHD2* overexpression was detected in the NTS regions as well (Figure 3C,D). Therefore, these results support the hypothesis that Jhd2 exerts its effects on all states of methylated H3K4 within the NTS regions of rDNA.

### Jhd2 regulates rDNA silencing through a Sir2-independent pathway

**Figure 4:**
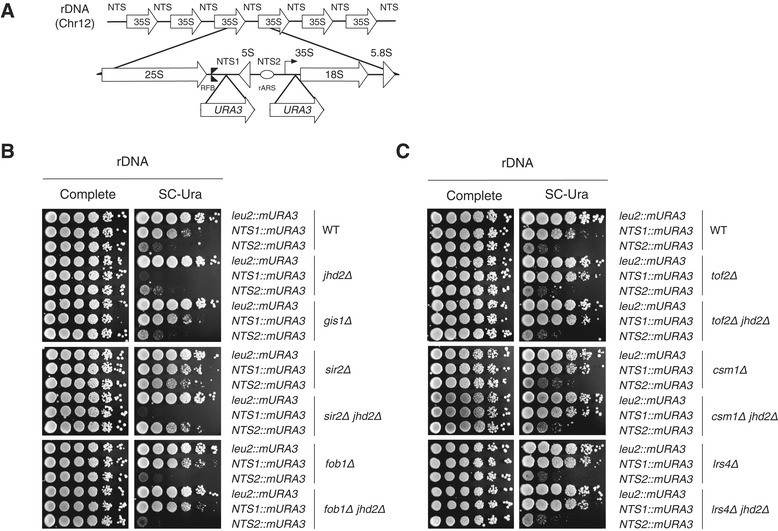
**Loss of Jhd2 enhances rDNA silencing at NTS1, but not NTS2, in a Sir2-independent manner. (A)** Schematic of an rDNA unit with the position of the *mURA3* reporters inserted into NTS1 or NTS2 (see Figure 2A for features). **(B, C)** *URA3*-based rDNA silencing assays at either NTS1 or NTS2. WT and the indicated deletion strains in the DMY2798 (*leu2::mURA3*), DMY2804 (*RDN1-NTS1::mURA3*) or DMY2800 (*RDN1-NTS2::mURA3*) background were used. Chr, chromosome; WT, wild type.

To establish further the role of Jhd2 in rDNA silencing, we used strains carrying an *mURA3* reporter gene integrated into one of three sites: outside the rDNA array at the *LEU2* gene or inside the rDNA unit at one of two loci with a strong signal for Net1 and Sir2 association (Figure 4A) [13]. Unlike the *mURA3* gene outside the rDNA, the *mURA3* gene inserted at either NTS1 or NTS2 exhibited strong silencing, which was compromised by *sir2Δ* in both cases*,* indicating that silencing at the NTS regions is influenced by Sir2 (Figure 4B, top and middle panes) [13]. The enhanced silencing at NTS2 correlated with increased enrichment of Net1 at chromatin near NTS2 (compare Figures 2C and 4B). By contrast, cells lacking Jhd2 displayed a profound increase in rDNA silencing, predominantly at NTS1, consistent with the ability of Jhd2 to modulate H3K4 methylation primarily at NTS1. Moreover, the *sir2Δ jhd2Δ* double mutation still significantly increased rDNA silencing at NTS1 to levels seen in *jhd2Δ* cells and decreased silencing at NTS2 to levels seen in *sir2Δ* cells. If the enhanced rDNA silencing at NTS1 caused by *jhd2Δ* was affected by Sir2, then deletion of *SIR2* should have at least partially rescued the growth defect observed in *jhd2Δ* cells (Figure 4B, top and middle panes). Silencing in *gis1Δ* cells was indistinguishable from that in WT cells. We therefore reasoned that Jhd2 regulates rDNA silencing specifically at the NTS1 region in a Sir2-independent manner.

We next examined whether proteins, such as Fob1, Tof2 and Csm1/Lrs4, are involved in regulating rDNA silencing by Jhd2. As previously reported, the deletion of *FOB1*, *TOF2*, *CSM1* or *LRS4* caused defects in NTS1-specific rDNA silencing (Figures 4B bottom pane and 4C) [14]. Intriguingly, we observed that cells containing a *JHD2* deletion combined with an *FOB1*, *TOF2*, *CSM1* or *LRS4* deletion grew at similar rate to *fob1Δ*, *tof2Δ*, *csm1Δ* or *lrs4Δ* cells (Figures 4B bottom pane and 4C). These results strongly suggest that Jhd2 regulates rDNA silencing through a pathway that includes Fob1, Tof2 and Csm1/Lrs4.

### Jhd2 regulates rDNA recombination through a Tof2/Csm1/Lrs4 pathway

**Figure 5:**
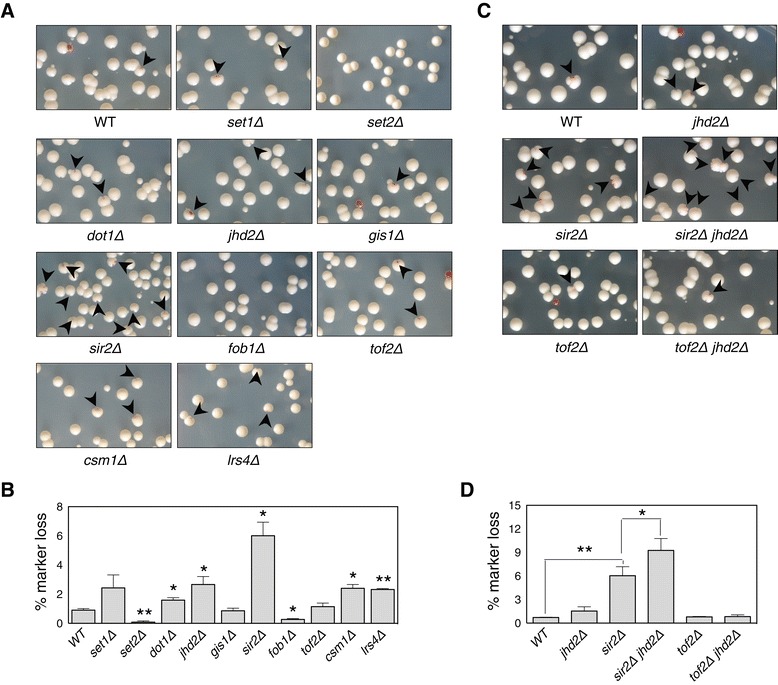
**Jhd2 regulates unequal sister chromatid exchange in rDNA in a Sir2- independent manner. (A, C)** The frequency of unequal rDNA crossovers was monitored by loss of the *ADE2* gene located within the rDNA array for WT (W303R) and the indicated deletion strains. **(B, D)** The percentage of *ADE2* gene loss (% marker loss) from **(A)** and **(C)** was calculated as the ratio of red-sectored colonies to the total number of colonies. Completely red colonies were excluded. Error bars indicate the SD from two **(B)** or three **(D)** repetitions, and asterisks indicate statistically significant differences compared with WT or between indicated pairs (*, *P* <0.05; **, *P* <0.01). WT, wild type.

In yeast, rDNA silencing within the nucleolus is thought to be part of a mechanism that evolved to suppress rDNA recombination among rDNA repeats. This repression of genome instability delays the formation of rDNA circles, the accumulation of which ultimately leads to cellular senescence [26,27]. Based on the finding that loss of H3K4 or H3K79 methylation resulted in disruption of RENT component recruitment to a silencing-compromised rDNA locus, we tested whether loss of Set1 or Dot1 affects rDNA recombination. To measure the rDNA recombination rate, we monitored the frequency of loss of an *ADE2* marker gene integrated at the rDNA locus, a technique known as the unequal sister chromatid exchange assay. As expected, we observed that the loss of Set1 or Dot1 led to approximately twofold increases in the rate of marker loss compared with WT cells (Figure 5A,B). By contrast, *ADE2* loss was completely suppressed in cells lacking Set2. As a control, we confirmed that marker loss was significantly elevated in *sir2Δ* cells, as previously reported [28]. Accordingly, these results demonstrate that the histone H3 methylases Set1, Dot1 and Set2 regulate rDNA recombination, consistent with their roles in Sir2-dependent rDNA silencing.

Considering that Jhd2 influences rDNA silencing independently of Sir2 but does associate with a set of proteins including Tof2/Csm1/Lrs4, we next asked whether Jhd2 regulates the rDNA recombination associated with these proteins. Consistent with previous reports showing that Fob1 is required for intra- and inter-sister chromatid recombination events following double-stranded breaks at the RFB site of rDNA [29,30], we observed that the rate of rDNA recombination was significantly reduced in *fob1Δ* cells (Figure 5A,B). However, for cells lacking either Csm1 or Lrs4, we observed a twofold increase in the rate of marker loss. Several reports have suggested that Csm1/Lrs4 regulates the rate of rDNA recombination through two possible models: Csm1/Lrs4 mediates the tethering of rDNA repeats to the nuclear periphery or recruits condensin to the RFB site of rDNA, either of which would help maintain the integrity of the rDNA repeat [14,31–33]. In cells lacking Tof2, we observed a more modest increase in marker loss than in cells lacking Csm1 or Lrs4, consistent with previous reports [14,33]. Collectively, these data support the idea that the proteins Fob1/Tof2/Csm1/Lrs4 help maintain the integrity of rDNA repeats.

We next hypothesized that if Jhd2 affects rDNA recombination through a Tof2/Csm1/Lrs4 pathway, loss of Jhd2 would most likely suppress rDNA recombination through enhanced rDNA silencing. Contrary to our expectation, deletion of *JHD2* caused a twofold increase in the rDNA recombination rate. Combining *jhd2Δ* with *sir2Δ* had an additive effect on the rate of rDNA recombination, whereas double mutant cells lacking *jhd2Δ tof2Δ*, *jhd2Δ csm1Δ* or *jhd2Δ lrs4Δ* did not show such an effect (Figure 5C,D and Additional file 1: Figure S4). Accordingly, these results demonstrate that Jhd2 contributes to the regulation of rDNA repeat integrity in a Sir2-independent manner, and they suggest that Jhd2 affects rDNA recombination through a Tof2/Csm1/Lrs4 pathway.

### Alleviation of condensin recruitment to rDNA by Jhd2

**Figure 6:**
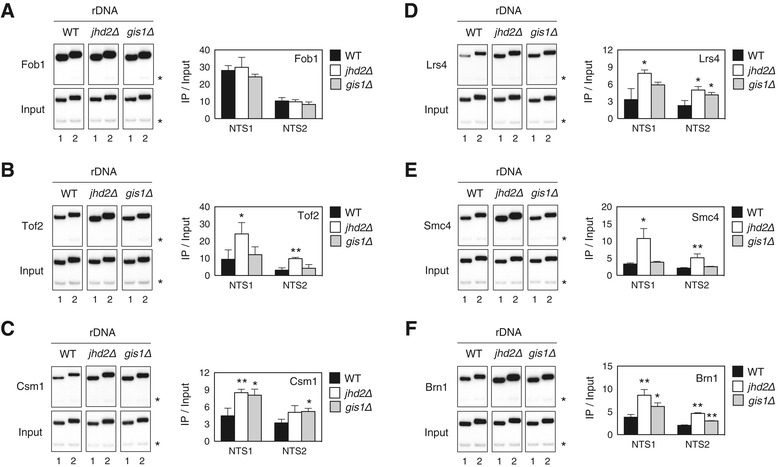
**Loss of Jhd2 increases association of condensin subunits, Tof2 and Csm1/Lrs4 with the RFB. (A-F)** ChIP analyses were performed using WT, *jhd2*Δ or *gis1*Δ strains carrying the indicated TAP-tagged proteins as shown in Figure 2B. Quantitation of each ChIP result is shown at the right. Error bars represent the SD of three PCRs performed using two independent chromatin preparations, and asterisks indicate statistically significant differences compared with WT (*, *P* <0.05; **, *P* <0.01). IP, immunoprecipitation; WT, wild type.

A recent study showed that the RFB site within the NTS1 region of the rDNA repeats functions as a *cis* element for Fob1-dependent recruitment of condensin to chromosomes [31]. The authors suggested that three proteins, Tof2, Csm1 and Lrs4, are required for the recruitment of condensin subunits, such as Smc4 or Brn1, to the RFB and that hierarchical binding of Fob1, Tof2, Csm1, Lrs4 and condensin helps maintain the integrity of long rDNA repeats. These findings raise the possibility that Jhd2 regulates silencing at the rDNA locus by recruiting condensin subunits. To test this hypothesis, ChIP was performed on asynchronous cultures of WT cells or cells lacking Jhd2 or Gis1, which expressed TAP-tagged Fob1, Tof2, Csm1 or Lrs4. Previous reports showed that the major peaks of Fob1, Tof2, Csm1 and Lrs4 binding overlap with NTS1, whereas smaller peaks were observed near NTS2 [13,14]. Similarly, we observed strong cross-linking of these four proteins at NTS1 and weak cross-linking at NTS2 (Figure 6A–D). Significantly, we found that loss of Jhd2 caused an approximately twofold increase in the association of Tof2/Csm1/Lrs4, but not Fob1, with the NTS regions, with greater concentrations of these proteins at NTS1 than at NTS2. Furthermore, we found that the *jhd2* mutation significantly increased the recruitment of Smc4 and Brn1 to the NTS regions, similar to the increase in Tof2/Csm1/Lrs4 recruitment (Figure 6E,F). In *gis1Δ* cells, the cross-linking of Fob1, Tof2 and Smc4 remained unchanged, whereas that of Csm1, Lrs4 and Brn1 was elevated at both NTS regions. Taken together, our results show that loss of Jhd2 significantly elevated the association of condensin subunits, Tof2 and Csm1/Lrs4 with NTS regions, and they strongly suggest that H3K4 demethylation by Jhd2 is a critical step in maintaining proper condensin association following Fob1 recruitment to the NTS1 region of rDNA repeats.

### Jhd2 regulates mitotic rDNA condensation

**Figure 7:**
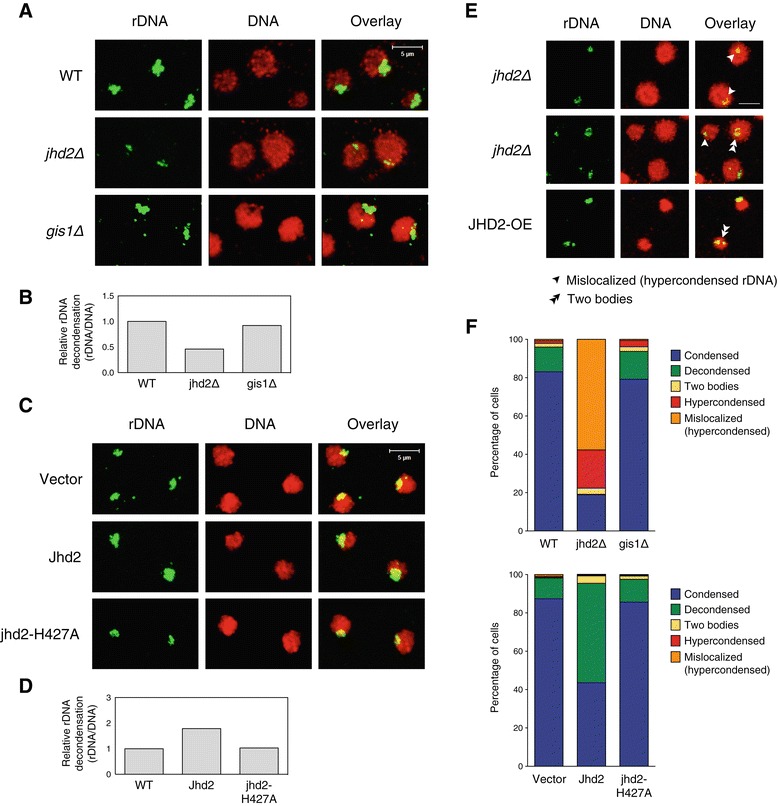
**Jhd2 regulates chromosome condensation at the rDNA locus. (A)** rDNA FISH was performed for WT, *jhd2*Δ or *gis1*Δ strains arrested at metaphase by treatment with nocodazole. Scale bar: 5 μm. **(B)** Quantitation of relative rDNA decondensation in **(A)**. The area of the green rDNA FISH signal was divided by the area of the red propidium iodide signal and then normalized to the WT value. **(C)** rDNA FISH was performed for cells containing empty vector (pRS424), constructs expressing HA-tagged WT Jhd2, or the H427A mutant, which were grown in SC medium lacking Trp. **(D)** Quantitation of relative rDNA decondensation in **(C)**. **(E)** Cells lacking or overexpressing Jhd2 used in **(A)** or **(C)** showing different rDNA morphologies (arrowhead, mislocalized and hypercondensed rDNA; double arrowhead, two bodies). **(F)** Quantitative analyses of rDNA morphology in cells used in **(A)** and **(C)**. Percentages of cells with the indicated rDNA phenotype are shown. WT, wild type.

Finally, we reasoned that if Jhd2 regulates rDNA silencing by affecting condensin recruitment, then the deletion or overexpression of *JHD2* should affect mitotic chromatin packaging, also referred to as mitotic rDNA condensation. To evaluate the input of Jhd2 in the establishment of mitotic chromatin changes, we monitored rDNA condensation in nocodazole-arrested cells by fluorescent *in situ* hybridization (FISH) [34]. We measured the ratio of the rDNA FISH signal from a probe against the highly repetitive rDNA array relative to total nuclear volume as an indication of nucleolar organization (relative rDNA decondensation ratio) [35]. We observed no differences in the relative rDNA decondensation ratio between WT and *gis1* mutant cells; however, the deletion of *JHD2* led to a more compact or hypercondensed rDNA morphology and a significant twofold decrease in the decondensation ratio compared with WT (Figure 7A,B). Approximately 77% of cells lacking Jhd2 contained hypercondensed rDNA (Figure 7F). Moreover, overexpression of *JHD2*, but not *GIS1*, caused the rDNA to adopt an amorphous distribution, which was reflected by an increase in the decondensation ratio up to 1.8-fold, whereas jhd2-H427A, a catalytically inactive mutant of Jhd2, had little effect on rDNA decondensation (Figure 7C,D and Additional file 1: Figure S3). Approximately 52% of cells overexpressing *JHD2* contained decondensed rDNA (Figure 7F). In particular, cell population analysis revealed that approximately 3.5% of *JHD2*-deficient or -overexpressing cells showed two separable rDNA bodies, whereas the majority (up to 70%) of *jhd2Δ* cells showed hypercondensed rDNA that was separated from the nuclear periphery (Figure 7E,F). Therefore, our FISH analyses demonstrate that histone H3K4 demethylation by Jhd2 regulates chromosome condensation at the rDNA locus during mitosis, and they suggest that Jhd2 is required for the sequestration of rDNA in the peripherally located nucleolus, a process that ensures rDNA repeat stability.

## Discussion

**Figure 8:**
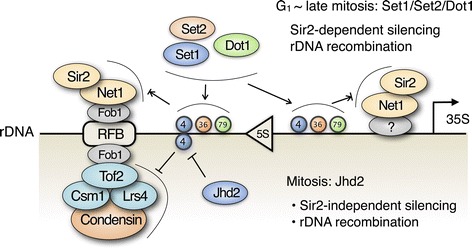
**Proposed model showing that nucleolar silencing is regulated by histone H3K4 demethylation by Jhd2.** One unit of the budding yeast rDNA is shown. The triangle and bent arrow indicate a 5S rRNA gene and the transcription start site for a 35S pre-rRNA gene, respectively, which are separated by two NTSs, NTS1 and NTS2. The numbers in colored circles represent methylated lysine residues on histone H3, namely methylated histone H3K4, H3K36 or H3K79. During the G_1_ to late mitotic phases, the histone H3 methylases Set1, Set2 and Dot1 primarily regulate rDNA recombination and nucleolar silencing in a Sir2-dependent manner; Set1 or Dot1 positively recruits the RENT components Net1 and Sir2 at both the NTS1 and NTS2 regions of the rDNA repeats, whereas Set2 has an opposing effect. During mitosis, however, both rDNA recombination and nucleolar silencing are regulated by the H3K4 demethylase Jhd2 in a Sir2-independent manner.

The results presented here illustrate the distinct role of histone H3 demethylation in regulating rDNA recombination and nucleolar silencing in yeast. Based on the results obtained in this study and other previous reports, we propose a model in which Jhd2 regulates rDNA silencing by maintaining proper condensin association with rDNA repeats, which contributes to faithful mitotic chromosome segregation (Figure 8). During interphase and mitosis, the RENT components Net1 and Sir2 colocalize to a subdomain within the nucleolus, particularly to the NTS1 and NTS2 rDNA regions; however, at the end of mitosis, Sir2 leaves the nucleolus and disperses throughout the nucleus [12,13]. The methylation of H3K4 and H3K79 by Set1 and Dot1, as well as the methylation of H3K36 by Set2, act as bivalent markers that affect Net1/Sir2 recruitment to both the NTS1 and NTS2 regions. The association of Net1/Sir2 with NTS1 depends on Fob1, but how Net1/Sir2 associates with NTS2 is still unknown. Changes in Net1/Sir2 recruitment to rDNA regulated by bivalent histone methylation may contribute to restricted nucleolar silencing, a paradoxical silencing of rDNA transcription in which silenced chromatin structures in the nucleolus allow for highly active transcription by RNA polymerase I [36]. In this context, it seems likely that rDNA packaging could dynamically shift between a Set1- or Dot1-engaged silenced structure and a Set2-engaged active structure accessible to RNA polymerase I. During mitosis, however, nucleolar silencing is likely to be primarily regulated by Jhd2, a histone H3K4 demethylase, in a Net1- or Sir2-independent manner. The yeast condensin localizes throughout the genome during interphase but becomes concentrated in the nucleolus during mitosis [35,37], and the RFB site within the NTS1 region functions as a *cis* element for Fob1-dependent condensin recruitment onto chromatin [31]. Demethylation of H3K4 by Jhd2, which occurs within a region of NTS1 that spans the RFB site, suppresses Fob1-mediated hierarchical binding of Tof2, Csm1 and Lrs4 to the RFB site, thereby limiting the excessive recruitment of condensin. This alleviation of condensin recruitment to the NTS1 region by Jhd2 is thought to ensure the proper condensin levels necessary to facilitate the higher-order structure of mitotic chromosomes, leading to faithful sister chromatid separation during anaphase. The prevention of excessive condensin recruitment to NTS1 by Jhd2 also contributes to nucleolar restriction during mitosis by allowing for less condensation of the chromatin in the nucleolus. Consistent with our model, the rRNA genes within the nucleoli of most mammalian cells contain more than 50% active NORs [8], which remain under-condensed during mitosis with a distinct decondensed chromatin structure [38].

Previously, it was reported that chromosomal regions harboring rDNA repeats are preferentially sequestered at the nuclear periphery away from the bulk DNA in yeast; however, deletion of *LRS4* or *HEH1*, a chromosome linkage inner nuclear membrane protein, caused the rDNA to adopt an amorphous distribution and reduced perinuclear rDNA positioning with a small percentage of cells showing two separable rDNA bodies [32,34]. Similarly, we observed that approximately 3.5% of *JHD2*-deficient or -overexpressing cells showed two separable rDNA bodies and the majority of the hypercondensed rDNA in *jhd2Δ* cells was mislocalized away from the nuclear periphery (Figure 7E,F). The presence of two separable rDNA bodies may reflect a strong loss of interactions between rDNA repeats on chromosome XII sister chromatids, as previously postulated in cells deficient for Lrs4, Csm1 or Heh1 [32]. In addition, a loss of rDNA sequestration in the peripherally located nucleolus in *jhd2Δ* cells is likely correlated with increased instability of the rDNA repeats, as a similar correlation was reported for *lrs4Δ* and *heh1Δ* cells [32]. This phenomenon could also explain the discrepancy between the increased rDNA silencing and the elevated rDNA recombination observed in *jhd2Δ* cells (see Figures 1G and 5B). For example, the increased Csm1/Lrs4 recruitment to the NTS1 region of the rDNA repeats in *jhd2Δ* cells could increase Csm1/Lrs4-dependent rDNA clustering to promote rDNA silencing, while simultaneously disrupting the stoichiometry of Csm1/Lrs4-dependent perinuclear rDNA anchoring, leading to mislocalization of rDNA at the nuclear periphery and increased rDNA instability. In contrast with the additive effect observed in the *jhd2Δ sir2Δ* double mutant, combinations of *jhd2Δ* with *csm1Δ* or *lrs4Δ* were indistinguishable from *jhd2Δ* alone, supporting the idea that the function of Jhd2 in regulating rDNA recombination is linked to that of Csm1 and Lrs4 (Additional file 1: Figure S4).

Recently, Csm1/Lrs4 proteins were shown to maintain replicative lifespan by regulating both rDNA repeats and telomeres [39]. At telomeric regions, the Csm1/Lrs4 proteins link inner nuclear proteins to chromosome ends, an interaction that requires the Sir proteins. Interestingly, the authors showed that deletion of *FOB1*, which recruits Csm1/Lrs4 to rDNA, increased the association of Lrs4 with DNA sequences 0.6 kb away from the telomeres, and they suggested that Lrs4 proteins released from rDNA relocate to available sites away from the telomeres. This model raises the possibility that the increased Csm1/Lrs4 recruitment to rDNA observed in *jhd2Δ* cells is an indirect effect of the release of a pool of Csm1/Lrs4 proteins from telomeres. If the increase in Csm1/Lrs4 association at rDNA regions in *jhd2Δ* cells is caused by the relocation of these proteins away from telomeres, a similar decrease in both Sir2 association and telomeric silencing, as seen in *lrs4Δ* cells, would be expected [39]. However, our group and Liang *et al*. observed that the loss of Jhd2 significantly enhanced telomeric silencing and increased recruitment of Sir2 proteins to telomere-proximal regions (Figure 1B,D,E) [5]. As such, it is unlikely that loss of Jhd2 caused a relocation of Csm1/Lrs4 proteins between the two silent chromatin domains.

It could be argued that Set1 opposes Jhd2 in regulating condensin recruitment to rDNA during mitosis based on the observation that overexpression of *JHD2* partially decreased all states of H3K4 methylation at the NTS regions of rDNA (Figure 3D). However, we believe that the ability of Jhd2 to modulate condensin recruitment is more likely to be distinct from that of Set1, as only H3K4me3 was significantly reduced, whereas H3K4me2 and H3K4me1 were only slightly affected by overexpression of *JHD2* (Figure 3D). Consistent with this idea, it was reported that H3K4me3 is enriched across most rDNA repeats, although this modification is increased 5- to 6-fold in *sir2Δ* cells, primarily at the NTS2 region [40]. Our results show that the increased rDNA silencing in *jhd2Δ* cells was independent of Sir2 and was only observed within the NTS1 but not the NTS2 region (Figure 4B). Therefore, a plausible explanation for these observations is that Set1-mediated H3K4 methylation is likely associated with rDNA silencing in a Sir2-dependent manner at both the NTS1 and NTS2 regions, whereas Jhd2-mediated H3K4 demethylation regulates rDNA silencing independently of Sir2 at the NTS1 region.

High-resolution mass spectrometry analyses suggested that Gis1 demethylates histone H3K36me2/1 [3]. Similarly, we observed that Gis1 has *in vivo* H3K36me2/1 demethylase activity at rDNA loci (Figure 3D and Additional file 1: Figure S2B). However, we found no evidence that Gis1 activity influences rDNA silencing. Two JmjC demethylases, Rph1 and Gis1, have been reported to regulate the expression of *PHR1*, a photolyase gene required for the light-dependent repair of pyrimidine dimers. Both demethylases contain two zinc fingers and are damage-responsive repressors of *PHR1* [41]. In other examples, Gis1 was suggested to act as a transcription factor regulating chronological lifespan; calorie restriction-dependent lifespan extension is mediated by the down-regulation of both the Ras and Tor-Sch9 pathways and the resulting activation of the Rim15-controlled Msn2/4 and Gis1. The activation of Gis1 then induces a variety of genes involved in G_0_ entry and the stress response [42–44]. It remains to be determined whether the role of Gis1 in regulating Net1 association with rDNA loci (Figure 2C) is linked to the function of Gis1 as a transcriptional repressor or activator. A previous report that the localization of Net1 to the NTS2 region may be due to its physical association with RNA polymerase I [13], raising the possibility that Gis1, at least in part, negatively regulates Net1 recruitment to the NTS2 region, presumably as a transcriptional repressor of RNA polymerase I. In addition, we do not dismiss the possibility that Gis1 may assist the Jhd2-mediated recruitment of condensin to rDNA, as we detected a considerable increase in the recruitment of Csm1, Lrs4 and Brn1 in *gis1Δ* cells (Figure 6C,D, F).

In vertebrate cells, there are two types of condensin complexes, condensin I and II [45]. These two complexes share two core subunits, CAP-E/SMC2 and CAP-C/SMC4, which belong to the structural maintenance of chromosomes (SMC) protein family; the complexes also contain three alternative non-SMC regulatory subunits, the kleisin subunit and two HEAT repeat-containing subunits (reviewed in [46]). In *S. cerevisiae*, the condensin complex is composed of five subunits: two core SMC subunits, Smc2 and Smc4, and three non-SMC subunits, Brn1 (kleisin subunit) and Ycs4/Ycs5 (HEAT repeat subunits) (reviewed in [47]).

As the SMC proteins are highly conserved from yeast to humans and Jhd2 is an evolutionarily conserved mammalian JARID1 family protein targeting histone H3K4, the regulation of rDNA condensation in mitosis by H3K4 demethylation is most likely conserved among eukaryotes.

## Conclusions

Nucleolar rDNA is tightly associated with heterochromatin. However, paradoxically, the compact structure of rDNA still allows for highly active transcription. In the present study, we show that rDNA packaging is dynamically regulated by changes in histone H3 lysine methylation levels between a Set1- or Dot1-engaged silenced structure and a Set2-engaged active structure accessible to RNA polymerase I, thereby affecting rDNA silencing and recombination between the rDNA repeats. During mitosis in particular, we found that the prevention of excessive condensin association within the NTS1 region by the Jhd2 demethylase contributes to the proper condensin level to facilitate the higher-order structure of mitotic chromosomes. The dynamic epigenetic changes in histone H3 methylation levels by histone H3 lysine methyltransferases and demethylases in the regulation of rDNA silencing provide a mechanistic insight regarding how restricted silencing within the nucleolus contributes to faithful chromosome segregation, as well as to cellular senescence during the progression of the cell cycle.

## Methods

### Strains and plasmids

The yeast strains and plasmids used in this study are listed in Additional file 1: Tables S1 and S2. The yeast transformations were performed using the standard lithium acetate method. To generate individual TAP-tagged and deletion strains, the C-terminal insertion cassette for TAP-tagging target genes and the disruption cassette for gene deletion were constructed by PCR amplification using genomic DNA from the corresponding strain from Open Biosystems or Euroscarf. The *JHD2* deletion strain (SY568) was directly generated by replacing the open reading frame of *JHD2* with the *HIS3MX6* module using the pFA6a-His3MX6 plasmid [48]. Strains containing substitution mutations at Lys4, Lys36 or Lys79 of histone H3 were generated by two different types of plasmid shuffling. The pMP9 (*LYS2 CEN ARS*) - *HHF2-HHT2* plasmid in the UCC1188 strain was replaced with the *TRP1*-based centromeric plasmids pWZ414-F13, pRS314-H3(K4A)-H4, pRS314-H3(K36A)-H4 or pWZ414-F13-H3(K79A)-H4 by counter-selection against the *LYS2* gene [49]. To select cells that lack the *LYS2* plasmid, cells were streaked on SC plates lacking tryptophan but containing 0.2% α-aminoadipic acid (US Biological). The UCC1111 strain was transformed with pRS314-H3(K4A)-H4 or pRS314-H3(K36A)-H4 and then selected on SC plates lacking tryptophan but containing 15 μM adenine six times until the WT plasmid, pRS412 (*ADE2 CEN ARS*) - *HHF2-HHT2*, was shuffled out and red colonies formed [50]. All strains were verified by PCR and/or Western blotting analysis.

### Growth conditions

Cells were grown at 30°C in YPD (1% yeast extract, 2% peptone and 2% glucose) or synthetic complete (SC) medium with the appropriate amino acids and bases, unless otherwise indicated. For *URA3*-based silencing assays or unequal sister chromatid exchange experiments, cells were grown to an A_600_ between 1.0 and 1.2 in YPD or SC medium, respectively. For ChIP experiments, all yeast strains were grown in SC medium to an A_600_ of 0.6, except for strains with the *URA3* reporter, which were cultured to an A_600_ of 0.1 in SC medium and then transferred to SC medium containing 0.1% FOA. For rDNA FISH analyses, cells grown to an A_600_ between 0.3 and 0.4 were treated with nocodazole for 2 hr at a final concentration of 15 μg/ml to yield cells arrested in metaphase. Galactose-inducible plasmids were induced in SC medium containing 2% galactose.

### Silencing assays

Telomeric silencing was measured using two different assays as described previously [51]. The colony color assay utilizes an *ADE2* reporter integrated next to the right telomere of chromosome V. Reporter strains were plated onto YPD medium, incubated for 2 days, and then transferred to 4°C until the color development was sufficient for photometric detection. *ade2* mutants accumulate a red pigment, a byproduct of an intermediate in the adenine biosynthesis pathway. Therefore, the cells turn red if *ADE2* is silenced, whereas they remain white if *ADE2* is not silenced. The switching of the *ADE2* reporters between the on and off states results in the characteristic sectored colonies. For *URA3*-based telomeric silencing assays, the cells were normalized to an A_600_ of 0.1 and then diluted with fivefold serial dilutions. Then 5 μl of each dilution was spotted onto SC medium or SC medium containing 0.1% FOA. The plates were incubated at 30°C for 2 or 3 days before imaging. The loss of silencing activates expression of *URA*3, preventing growth on FOA. Silencing at the silent mating loci (*HMR* and *HML)* and rDNA was analyzed in the same manner, with the exception that cells were grown to an A_600_ of 1.0 and plated onto SC medium, SC medium lacking uracil or SC medium containing FOA. Cells lacking *LRS4* or *CSM1* were spotted in parallel with WT cells but were photographed later because the two types of mutant cells grow more slowly than WT cells [52].

### Chromatin immunoprecipitation

ChIP experiments were performed as described previously [52]. The oligonucleotide sequences used in ChIP PCR are listed in Additional file 1: Table S3. Briefly, TAP-tagged proteins were precipitated with 20 μl of IgG-Sepharose beads (GE Healthcare). The antibodies listed in Additional file 1: Table S4 were bound to 10 μl of protein A-Sepharose CL-4B (GE Healthcare) to precipitate chromatin. Quantitation of the PCR signals was performed using the image analyzer BAS1500 (Fuji). To control for the amplification efficiency and label incorporation of different primers, the PCR signals were normalized to the internal control and the input DNA. The results for methylated H3 were further normalized to total histone H3 signals. For Figure 3A and Additional file 1: Figure S2A, 21 PCR amplification cycles were carried out with diluted template DNA (1:5 dilution for the immunoprecipitated DNA, 1:200 dilution for the input DNA) to optimize conditions within the linear range of the exponential PCR amplification curve, as described previously [53]. The PCR signals were normalized to total H3 signal, and the results are presented as fold enrichment relative to WT.

### Preparation of yeast whole cell extracts and immunoblotting

Whole yeast cell extracts were prepared as described previously [52]. Immunoblotting analysis was performed using anti-HA, anti-H3K4me3 and anti-histone H3 antibodies (Additional file 1: Tables S4).

### Unequal sister chromatid exchange assays

The rate of marker loss through unequal recombination of an *ADE2* marker inserted into the rDNA array was measured as described previously [54]. Cells grown to an A_600_ of 1.0 were plated at a density of approximately 400 cells per SC plate containing a low concentration of adenine (27 μM) [53]. The plates were incubated at 30°C for 2 or 3 days and then transferred to 4°C for 2 weeks to enhance development of the red color. Sectors that lost the *ADE2* reporter from the rDNA array turned red due to the accumulation of an intermediate in the adenine biosynthesis pathway. Approximately 12,000 colonies counted using the Gene Tools software (Syngene) were used for each unequal sister chromatid exchange assay. The percentage of marker loss was calculated by dividing the number of red-sectored colonies by the total number of colonies. Completely red colonies indicate marker loss before plating and were excluded.

### Fluorescent *in situ* hybridization

FISH experiments were performed as described previously [34]. To prepare rDNA probes, *Bgl II* fragments from the plasmid p362, which contains the 5′ half of an rDNA unit (18S IVS, 5.8S IVS and the 5′ end of 25S), were labeled using the DIG Nick Translation Mix (Roche). Cells were arrested by treatment with nocodazole and used for FISH analysis. Hybridized rDNA probes were detected by serial incubations with 1:250 dilutions of anti-DIG mouse monoclonal IgG, luorescein isothiocyanate (FITC)-conjugated goat anti-mouse IgG and FITC-conjugated donkey anti-goat IgG. Bulk DNA was stained with propidium iodide to visualize total chromosomal DNA. Z-stack images at 0.15-μm intervals were acquired using a C-Apochromat 63x/1.20 W Corr M27 objective on the Zeiss LSM 700 confocal microscope, from which a three-dimensional reconstruction was generated using the method of maximum intensity projection. Image capture, analysis and processing were carried out using ZEN 2009 software (Carl Zeiss). The rDNA FISH signal was quantified using the segmentation function of IP Lab (Scanalytics). The area of the green rDNA FISH signal was divided by the area of the red propidium iodide signal and further normalized to the WT value. For each FISH experiment shown in Figure 7A,C, and Additional file 1: Figure S3A, a total of 400, 280 and 250 nuclei were analyzed, respectively.

## Additional file

Additional file 1:
**Supplementary information.** This file contains **Figures S1–S4** and **Tables S1–S4**.

## Abbreviations

bp: base pair
ChIP: chromatin immunoprecipitation
FISH: fluorescent *in situ* hybridization
FOA: 5-fluoroorotic acid
JHDM: Jumonji C domain-containing histone demethylases
JmjC: Jumonji C
IgG: immunoglobulin G
IP: immunoprecipitation
kb: kilobase
NOR: nucleolar organizer region
NTS: nontranscribed spacer
PCR: polymerase chain reaction
RENT: regulator of nucleolar silencing and telophase exit
RFB: replication fork barrier
SC: synthetic complete
SD: standard deviation
SMC: structural maintenance of chromosomes family
WT: wild type
