# Supplementary material for: Yeast histone H3 lysine 4 demethylase Jhd2 regulates mitotic ribosomal DNA condensation
Source: BMC Biol. 2014 Sep 24;12:75. doi: 10.1186/s12915-014-0075-3 (PMC4201760; doi:10.1186/s12915-014-0075-3)
Supplement: Additional file 1: — Supplementary information. This file contains Figures S1–S4 and Tables S1–S4. [file 12915_2014_75_MOESM1_ESM.pdf]

Ryu and Ahn, Figure S1

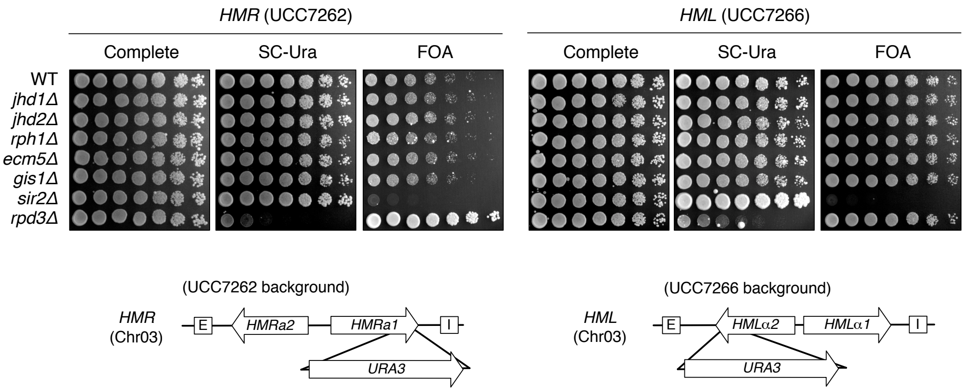

Figure S1 Deletion of each JmjC demethylase had no effect on the silencing at *HM* loci. *URA3*-based silencing assays at regions within *HMR* and *HML* loci were performed in WT and the indicated mutant strains in the UCC7262 and UCC7266 backgrounds. Schematic diagrams of the *URA3* reporter for each assay are shown below each panel.

Ryu and Ahn, Figure S2

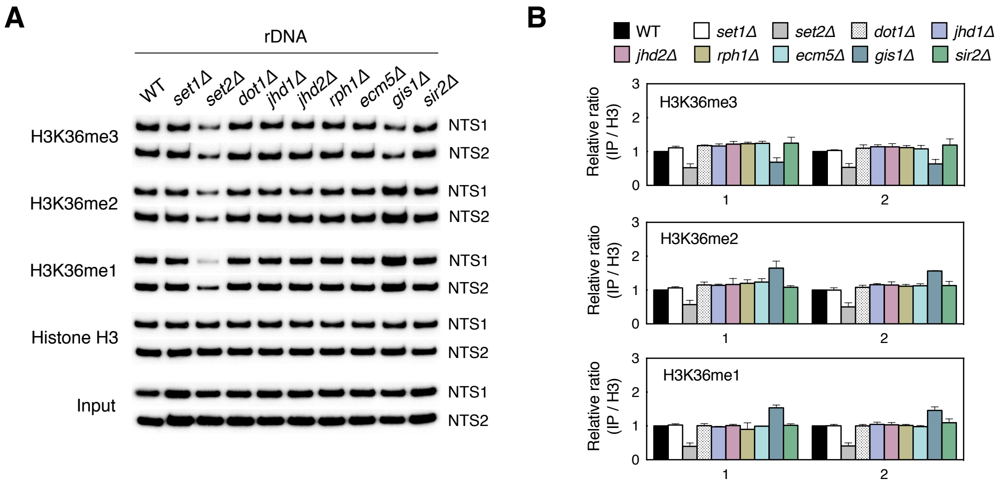

Figure S2 Gis1 demethylates H3K36 within the rDNA regions *in vivo*. (A) The levels of histone H3K36 methylation were analyzed by ChIP in the indicated strains. Antibodies against H3K36me3, H3K36me2, H3K36me1 or H3 were used. The input DNA and H3 bands are the same as in Figure 3A. **(B)** Quantitation of the ChIP results in **A**. The results for methyl-H3 were normalized to the total H3 signal and presented as fold enrichment relative to WT. Error bars indicate the S.D. from three PCRs performed using two independent chromatin preparations.

Ryu and Ahn, Figure S3

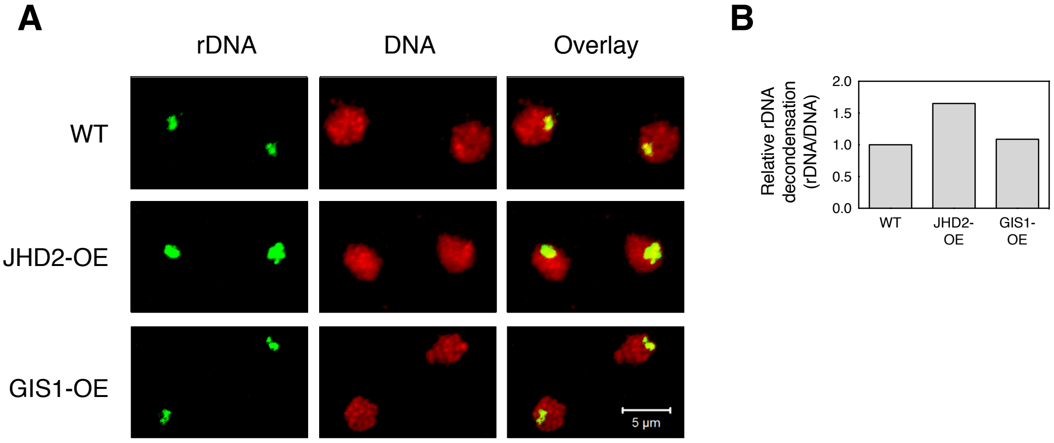

Figure S3 Overexpression of *JHD2* caused the rDNA to adopt an amorphous or decondensed distribution (A) rDNA FISH was performed in cells containing pRS325-GALpro (Vector), pRS325-GALpro-Jhd2-HA (JHD2-OE), or pRS325-GALpro-Gis1-HA (GIS1-OE). Cells were arrested at metaphase by treatment with nocodazole. Bar, 5 μm. **(B)** The quantitation results of relative rDNA decondensation are shown. The area of green rDNA FISH signal was divided by the red propidium iodide signal and further normalized to the WT value.

Ryu and Ahn, Figure S4

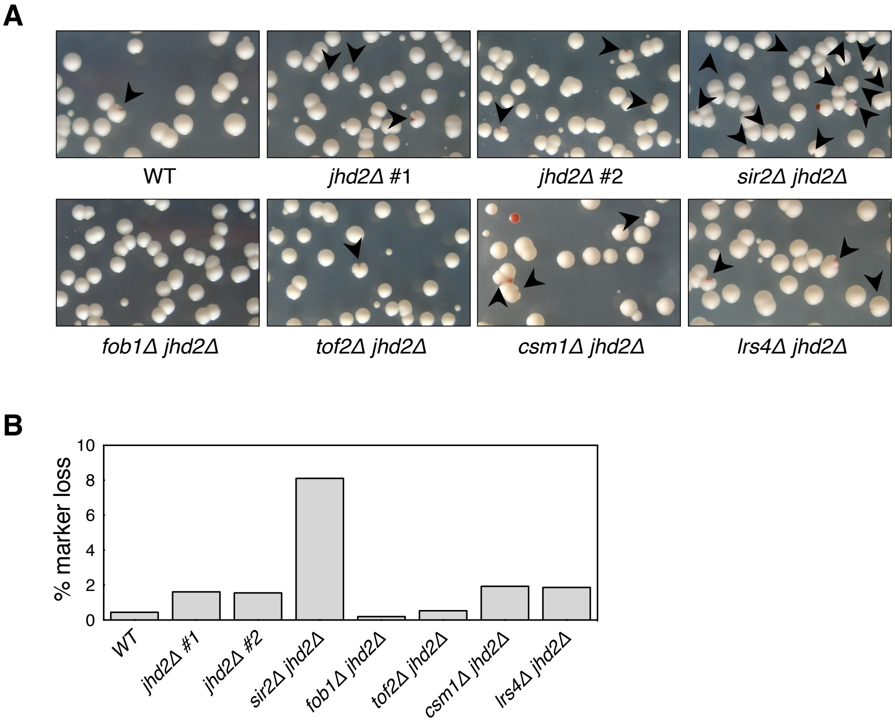

Figure S4 Jhd2 regulates unequal sister chromatid exchange in rDNA in a Csm1/Lrs4-dependent manner. (A) The frequency of unequal rDNA crossovers was monitored by loss of the *ADE2* gene located within the rDNA array in WT (W303R) and the indicated deletion strains as shown in Figure 5A. (B) The percent of *ADE2* gene loss (% marker loss) from A was calculated as the ratio of red-sectored colonies to the total number of colonies. Completely red colonies were excluded.

Table S1 Strains used in this study.

Figure	Strain	Genotype	Source	Note (Reference)
Figure 1B	BY4709	MATα ura3Δ0	Fred van Leeuwen	[1]
	BY4725	MATa ura3Δ0 ade2Δ::hisG	Daniel E. Gottschling	[2]
	UCC4825	MATa ura3Δ0 ade2Δ::hisG ADE2-TEL-VR	Daniel E. Gottschling	[2]
	SY266	MATa ura3Δ0 ade2Δ::hisG ADE2-TEL-VR sir2Δ::KanMX4	This study	From UCC4825
	SY267	MATa ura3Δ0 ade2Δ::hisG ADE2-TEL-VR jhd1Δ::KanMX4	This study	From UCC4825
	SY268	MATa ura3Δ0 ade2Δ::hisG ADE2-TEL-VR jhd2Δ::KanMX4	This study	From UCC4825
	SY269	MATa ura3Δ0 ade2Δ::hisG ADE2-TEL-VR rph1Δ::KanMX4	This study	From UCC4825
	SY270	MATa ura3Δ0 ade2Δ::hisG ADE2-TEL-VR ecm5Δ::KanMX4	This study	From UCC4825
	SY271	MATa ura3Δ0 ade2Δ::hisG ADE2-TEL-VR gis1Δ::KanMX4	This study	From UCC4825
Figure 1D	UCC1111	MATa ura3Δ0 leu2Δ0 trp1Δ63 his3Δ200 ade2Δ::hisG lys2Δ0 met15Δ0 adh4::URA3-TEL (VII-L) hhf2-hht2::MET15 hhf1-hht1::LEU2 pRS412 (ADE2 CEN ARS) - HHF2-HHT2	Kevin Struhl & Mark R. Parthun	[3,4]
	SY100	MATa ura3Δ0 leu2Δ0 trp1Δ63 his3Δ200 ade2Δ::hisG lys2Δ0 met15Δ0 adh4::URA3-TEL (VII-L) hhf2-hht2::MET15 hhf1-hht1::LEU2 jhd1Δ::KanMX4 pRS412 (ADE2 CEN ARS) - HHF2-HHT2	This study	From UCC1111
	SY101	MATa ura3Δ0 leu2Δ0 trp1Δ63 his3Δ200 ade2Δ::hisG lys2Δ0 met15Δ0 adh4::URA3-TEL (VII-L) hhf2-hht2::MET15 hhf1-hht1::LEU2 jhd2Δ::KanMX4 pRS412 (ADE2 CEN ARS) - HHF2-HHT2	This study	From UCC1111
	SY104	MATa ura3Δ0 leu2Δ0 trp1Δ63 his3Δ200 ade2Δ::hisG lys2Δ0 met15Δ0 adh4::URA3-TEL (VII-L) hhf2-hht2::MET15 hhf1-hht1::LEU2 rph1Δ::KanMX4 pRS412 (ADE2 CEN ARS) - HHF2-HHT2	This study	From UCC1111
	SY102	MATa ura3Δ0 leu2Δ0 trp1Δ63 his3Δ200 ade2Δ::hisG lys2Δ0 met15Δ0 adh4::URA3-TEL (VII-L) hhf2-hht2::MET15 hhf1-hht1::LEU2 ecm5Δ::KanMX4 pRS412 (ADE2 CEN ARS) - HHF2-HHT2	This study	From UCC1111
	SY103	MATa ura3Δ0 leu2Δ0 trp1Δ63 his3Δ200 ade2Δ::hisG lys2Δ0 met15Δ0 adh4::URA3-TEL (VII-L) hhf2-hht2::MET15 hhf1-hht1::LEU2 gis11Δ::KanMX4 pRS412 (ADE2 CEN ARS) - HHF2-HHT2	This study	From UCC1111
	SY238	MATa ura3Δ0 leu2Δ0 trp1Δ63 his3Δ200 ade2Δ::hisG lys2Δ0 met15Δ0 adh4::URA3-TEL (VII-L) hhf2-hht2::MET15 hhf1-hht1::LEU2 sir2Δ::KanMX4 pRS412 (ADE2 CEN ARS) - HHF2-HHT2	[5]	From UCC1111
	SY365	MATa ura3Δ0 leu2Δ0 trp1Δ63 his3Δ200 ade2Δ::hisG lys2Δ0 met15Δ0 adh4::URA3-TEL (VII-L) hhf2-hht2::MET15 hhf1-hht1::LEU2 rpd3Δ::KanMX6 pRS412 (ADE2 CEN ARS) - HHF2-HHT2	[5]	From UCC1111
Figure 1E	UCC1111	See Figure 1D		
	SY100	See Figure 1D		
	SY101	See Figure 1D		
	SY104	See Figure 1D		
	SY102	See Figure 1D		
	SY103	See Figure 1D		
Figure 1G	UCC1188	MATα ura3(-52 or -167) leu2Δ1 trp1(Δ901 or ::HIS3) his3Δ200 lys2-801 RDN1::URA3 hhf2-hht2::HIS3 hhf1-hht1::LEU2 pMP9 (LYS2 CEN ARS) - HHF2-HHT2	Daniel E. Gottschling	[6]
	SY477	MATα ura3(-52 or -167) leu2Δ1 trp1(Δ901 or ::HIS3) his3Δ200 lys2-801 RDN1::URA3 hhf2-hht2::HIS3 hhf1-hht1::LEU2 jhd1Δ::KanMX4 pMP9 (LYS2 CEN ARS) -	This study	From UCC1188

		HHF2-HHT2		
	SY382	MATα ura3(-52 or -167) leu2Δ1 trp1(Δ901 or ::HIS3) his3Δ200 lys2-801 RDN1::URA3 hhf2-hht2::HIS3 hhf1-hht1::LEU2 jhd2Δ::KanMX4 pMP9 (LYS2 CEN ARS) - HHF2-HHT2	This study	From UCC1188
	SY478	MATα ura3(-52 or -167) leu2Δ1 trp1(Δ901 or ::HIS3) his3Δ200 lys2-801 RDN1::URA3 hhf2-hht2::HIS3 hhf1-hht1::LEU2 rph1Δ::KanMX4 pMP9 (LYS2 CEN ARS) - HHF2-HHT2	This study	From UCC1188
	SY479	MATα ura3(-52 or -167) leu2Δ1 trp1(Δ901 or ::HIS3) his3Δ200 lys2-801 RDN1::URA3 hhf2-hht2::HIS3 hhf1-hht1::LEU2 ecm5Δ::KanMX4 pMP9 (LYS2 CEN ARS) - HHF2-HHT2	This study	From UCC1188
	SY480	MATα ura3(-52 or -167) leu2Δ1 trp1(Δ901 or ::HIS3) his3Δ200 lys2-801 RDN1::URA3 hhf2-hht2::HIS3 hhf1-hht1::LEU2 gis1Δ::KanMX4 pMP9 (LYS2 CEN ARS) - HHF2-HHT2	This study	From UCC1188
Figure 1H and 1I	SY702	MATα ura3(-52 or -167) leu2Δ1 trp1(Δ901 or ::HIS3) his3Δ200 lys2-801 RDN1::URA3 hhf2-hht2::HIS3 hhf1-hht1::LEU2 pMP9 (LYS2 CEN ARS) - HHF2-HHT2 pRS424	This study	From UCC1188
	SY703 [a]	MATα ura3(-52 or -167) leu2Δ1 trp1(Δ901 or ::HIS3) his3Δ200 lys2-801 RDN1::URA3 hhf2-hht2::HIS3 hhf1-hht1::LEU2 pMP9 (LYS2 CEN ARS) - HHF2-HHT2 pRS424-YJR119c-3XHA	This study	From UCC1188
	SY704 [a]	MATα ura3(-52 or -167) leu2Δ1 trp1(Δ901 or ::HIS3) his3Δ200 lys2-801 RDN1::URA3 hhf2-hht2::HIS3 hhf1-hht1::LEU2 pMP9 (LYS2 CEN ARS) - HHF2-HHT2 pRS424-yjr119c-3XHA(H427A)	This study	From UCC1188
Figure 2B	FY366	MATa ura3Δ0 leu2Δ0 his3Δ1 met15Δ0 NET1-TAP tag::HIS3MX6	Euroscarf	
	SY482	MATa ura3Δ0 leu2Δ0 his3Δ1 met15Δ0 set1Δ::KanMX4 NET1-TAP tag::HIS3MX6	This study	
	SY483	MATa ura3Δ0 leu2Δ0 his3Δ1 met15Δ0 set2Δ::KanMX4 NET1-TAP tag::HIS3MX6	This study	
	SY484	MATa ura3Δ0 leu2Δ0 his3Δ1 met15Δ0 dot1Δ::KanMX4 NET1-TAP tag::HIS3MX6	This study	
	SY485	MATa ura3Δ0 leu2Δ0 his3Δ1 met15Δ0 jhd1Δ::KanMX4 NET1-TAP tag::HIS3MX6	This study	
	SY486	MATa ura3Δ0 leu2Δ0 his3Δ1 met15Δ0 jhd2Δ::KanMX4 NET1-TAP tag::HIS3MX6	This study	
	SY487	MATa ura3Δ0 leu2Δ0 his3Δ1 met15Δ0 rph1Δ::KanMX4 NET1-TAP tag::HIS3MX6	This study	
	SY488	MATa ura3Δ0 leu2Δ0 his3Δ1 met15Δ0 ecm5Δ::KanMX4 NET1-TAP tag::HIS3MX6	This study	
	SY489	MATa ura3Δ0 leu2Δ0 his3Δ1 met15Δ0 gis1Δ::KanMX4 NET1-TAP tag::HIS3MX6	This study	
	SY490	MATa ura3Δ0 leu2Δ0 his3Δ1 met15Δ0 sir2Δ::KanMX4 NET1-TAP tag::HIS3MX6	This study	
Figure 2D	SY537	MATα ura3(-52 or -167) leu2Δ1 trp1(Δ901 or ::HIS3) his3Δ200 lys2-801 RDN1::URA3 hhf2-hht2::HIS3 hhf1-hht1::LEU2 pWZ414-F13	This study	From UCC1188
	SY538	MATα ura3(-52 or -167) leu2Δ1 trp1(Δ901 or ::HIS3) his3Δ200 lys2-801 RDN1::URA3 hhf2-hht2::HIS3 hhf1-hht1::LEU2 pRS314-H3(K4A)-H4	This study	From UCC1188
	SY539	MATα ura3(-52 or -167) leu2Δ1 trp1(Δ901 or ::HIS3) his3Δ200 lys2-801 RDN1::URA3 hhf2-hht2::HIS3 hhf1-hht1::LEU2 pRS314-H3(K36A)-H4	This study	From UCC1188
	SY540	MATα ura3(-52 or -167) leu2Δ1 trp1(Δ901 or ::HIS3) his3Δ200 lys2-801 RDN1::URA3 hhf2-hht2::HIS3 hhf1-hht1::LEU2 pWZ414-F13-H3(K79A)-H4	This study	From UCC1188
Figure 2E	SY507	MATa ura3Δ0 leu2Δ0 trp1Δ63 his3Δ200 ade2Δ::hisG lys2Δ0 met15Δ0 adh4::URA3-TEL (VII-L) hhf2-hht2::MET15 hhf1-hht1::LEU2 NET1-TAP tag::HIS3MX6 pRS412 (ADE2 CEN ARS) - HHF2-HHT2	This study	From UCC1111
	SY508	MATa ura3Δ0 leu2Δ0 trp1Δ63 his3Δ200 ade2Δ::hisG lys2Δ0 met15Δ0 adh4::URA3-TEL (VII-L) hhf2-	This study	From UCC1111

		hht2::MET15 hhf1-hht1::LEU2 NET1-TAP tag::HIS3MX6 pRS314-H3(K4A)-H4		
	SY509	*MATa ura3Δ0 leu2Δ0 trp1Δ63 his3Δ200 ade2Δ::hisG lys2Δ0 met15Δ0 adh4::URA3*-TEL (VII-L) *hhf2-hht2::MET15 hhf1-hht1::LEU2 NET1-TAP tag::HIS3MX6* pRS314-H3(K36A)-H4	This study	From UCC1111
	SY510	*MATa ura3Δ0 leu2Δ0 trp1Δ63 his3Δ200 ade2Δ::hisG lys2Δ0 met15Δ0 adh4::URA3*-TEL (VII-L) *hhf2-hht2::MET15 hhf1-hht1::LEU2 NET1-TAP tag::HIS3MX6* pF13 (*TRP1 CEN ARS*) - *hht2K79A-HHF2*	This study	From UCC1111
Figure 3A	FY366	See Figure 2B		
	SY482	See Figure 2B		
	SY483	See Figure 2B		
	SY484	See Figure 2B		
	SY485	See Figure 2B		
	SY486	See Figure 2B		
	SY487	See Figure 2B		
	SY488	See Figure 2B		
	SY489	See Figure 2B		
	SY490	See Figure 2B		
Figure 3C	SY565	*MATa ura3Δ0 leu2Δ0 his3Δ1 met15Δ0* pRS325-GALpro	This study	
	SY566[a]	*MATa ura3Δ0 leu2Δ0 his3Δ1 met15Δ0* pRS325-GALpro-YJR119c-3XHA	This study	
	SY567	*MATa ura3Δ0 leu2Δ0 his3Δ1 met15Δ0* pRS325-GALpro-GIS1-3XHA	This study	
Figure 4B	DMY2798	*MATa ura3-1 leu2-3,112 trp1-1 his3-11,15 ade2-1 can1-100 leu2::mURA3*	Won-Ki Huh & Danesh Moazed	[7,8]
	DMY2804	*MATa ura3-1 leu2-3,112 trp1-1 his3-11,15 ade2-1 can1-100 RDN1-NTS1::mURA3*	Won-Ki Huh & Danesh Moazed	[7,8]
	DMY2800	*MATa ura3-1 leu2-3,112 trp1-1 his3-11,15 ade2-1 can1-100 RDN1-NTS2::mURA3*	Won-Ki Huh & Danesh Moazed	[7,8]
	SY617	*MATa ura3-1 leu2-3,112 trp1-1 his3-11,15 ade2-1 can1-100 leu2::mURA3 jhd2Δ::KanMX4*	This study	From DMY2798
	SY620	*MATa ura3-1 leu2-3,112 trp1-1 his3-11,15 ade2-1 can1-100 RDN1-NTS1::mURA3 jhd2Δ::KanMX4*	This study	From DMY2804
	SY623	*MATa ura3-1 leu2-3,112 trp1-1 his3-11,15 ade2-1 can1-100 RDN1-NTS2::mURA3 jhd2Δ::KanMX4*	This study	Form DMY2800
	SY618	*MATa ura3-1 leu2-3,112 trp1-1 his3-11,15 ade2-1 can1-100 leu2::mURA3 gis1Δ::KanMX4*	This study	From DMY2798
	SY621	*MATa ura3-1 leu2-3,112 trp1-1 his3-11,15 ade2-1 can1-100 RDN1-NTS1::mURA3 gis1Δ::KanMX4*	This study	From DMY2804
	SY624	*MATa ura3-1 leu2-3,112 trp1-1 his3-11,15 ade2-1 can1-100 RDN1-NTS2::mURA3 gis1Δ::KanMX4*	This study	Form DMY2800
	SY579	*MATa ura3-1 leu2-3,112 trp1-1 his3-11,15 ade2-1 can1-100 leu2::mURA3 sir2Δ::KanMX4*	This study	From DMY2798
	SY584	*MATa ura3-1 leu2-3,112 trp1-1 his3-11,15 ade2-1 can1-100 RDN1-NTS1::mURA3 sir2Δ::KanMX4*	This study	From DMY2804
	SY589	*MATa ura3-1 leu2-3,112 trp1-1 his3-11,15 ade2-1 can1-100 RDN1-NTS2::mURA3 sir2Δ::KanMX4*	This study	Form DMY2800
	SY632[b]	*MATa ura3-1 leu2-3,112 trp1-1 his3-11,15 ade2-1 can1-100 leu2::mURA3 sir2Δ::KanMX4 jhd2Δ::HIS3MX6*	This study	From DMY2798
	SY638[b]	*MATa ura3-1 leu2-3,112 trp1-1 his3-11,15 ade2-1 can1-100 RDN1-NTS1::mURA3 sir2Δ::KanMX4 jhd2Δ::HIS3MX6*	This study	From DMY2804
	SY644[b]	*MATa ura3-1 leu2-3,112 trp1-1 his3-11,15 ade2-1 can1-100 RDN1-NTS2::mURA3 sir2Δ::KanMX4 jhd2Δ::HIS3MX6*	This study	Form DMY2800
	SY602	*MATa ura3-1 leu2-3,112 trp1-1 his3-11,15 ade2-1 can1-100 leu2::mURA3 fob1Δ::KanMX4*	This study	From DMY2798
	SY605	*MATa ura3-1 leu2-3,112 trp1-1 his3-11,15 ade2-1 can1-100 RDN1-NTS1::mURA3 fob1Δ::KanMX4*	This study	From DMY2804

	SY608	*MATa ura3-1 leu2-3,112 trp1-1 his3-11,15 ade2-1 can1-100 RDN1-NTS2::mURA3 fob1Δ::KanMX4*	This study	Form DMY2800
	SY633[b]	*MATa ura3-1 leu2-3,112 trp1-1 his3-11,15 ade2-1 can1-100 leu2::mURA3 fob1Δ::KanMX4 jhd2Δ::HIS3MX6*	This study	From DMY2798
	SY639[b]	*MATa ura3-1 leu2-3,112 trp1-1 his3-11,15 ade2-1 can1-100 RDN1-NTS1::mURA3 fob1Δ::KanMX4 jhd2Δ::HIS3MX6*	This study	From DMY2804
	SY645[b]	*MATa ura3-1 leu2-3,112 trp1-1 his3-11,15 ade2-1 can1-100 RDN1-NTS2::mURA3 fob1Δ::KanMX4 jhd2Δ::HIS3MX6*	This study	Form DMY2800
Figure 4C	DMY2798	See Figure 4B		
	DMY2804	See Figure 4B		
	DMY2800	See Figure 4B		
	SY616	*MATa ura3-1 leu2-3,112 trp1-1 his3-11,15 ade2-1 can1-100 leu2::mURA3 tof2Δ::KanMX4*	This study	From DMY2798
	SY619	*MATa ura3-1 leu2-3,112 trp1-1 his3-11,15 ade2-1 can1-100 RDN1-NTS1::mURA3 tof2Δ::KanMX4*	This study	From DMY2804
	SY622	*MATa ura3-1 leu2-3,112 trp1-1 his3-11,15 ade2-1 can1-100 RDN1-NTS2::mURA3 tof2Δ::KanMX4*	This study	Form DMY2800
	SY634[b]	*MATa ura3-1 leu2-3,112 trp1-1 his3-11,15 ade2-1 can1-100 leu2::mURA3 tof2Δ::KanMX4 jhd2Δ::HIS3MX6*	This study	From DMY2798
	SY640[b]	*MATa ura3-1 leu2-3,112 trp1-1 his3-11,15 ade2-1 can1-100 RDN1-NTS1::mURA3 tof2Δ::KanMX4 jhd2Δ::HIS3MX6*	This study	From DMY2804
	SY646[b]	*MATa ura3-1 leu2-3,112 trp1-1 his3-11,15 ade2-1 can1-100 RDN1-NTS2::mURA3 tof2Δ::KanMX4 jhd2Δ::HIS3MX6*	This study	Form DMY2800
	SY603	*MATa ura3-1 leu2-3,112 trp1-1 his3-11,15 ade2-1 can1-100 leu2::mURA3 csm1Δ::KanMX4*	This study	From DMY2798
	SY606	*MATa ura3-1 leu2-3,112 trp1-1 his3-11,15 ade2-1 can1-100 RDN1-NTS1::mURA3 csm1Δ::KanMX4*	This study	From DMY2804
	SY609	*MATa ura3-1 leu2-3,112 trp1-1 his3-11,15 ade2-1 can1-100 RDN1-NTS2::mURA3 csm1Δ::KanMX4*	This study	Form DMY2800
	SY635[b]	*MATa ura3-1 leu2-3,112 trp1-1 his3-11,15 ade2-1 can1-100 leu2::mURA3 csm1Δ::KanMX4 jhd2Δ::HIS3MX6*	This study	From DMY2798
	SY641[b]	*MATa ura3-1 leu2-3,112 trp1-1 his3-11,15 ade2-1 can1-100 RDN1-NTS1::mURA3 csm1Δ::KanMX4 jhd2Δ::HIS3MX6*	This study	From DMY2804
	SY647[b]	*MATa ura3-1 leu2-3,112 trp1-1 his3-11,15 ade2-1 can1-100 RDN1-NTS2::mURA3 csm1Δ::KanMX4 jhd2Δ::HIS3MX6*	This study	Form DMY2800
	SY604	*MATa ura3-1 leu2-3,112 trp1-1 his3-11,15 ade2-1 can1-100 leu2::mURA3 lrs4Δ::KanMX4*	This study	From DMY2798
	SY607	*MATa ura3-1 leu2-3,112 trp1-1 his3-11,15 ade2-1 can1-100 RDN1-NTS1::mURA3 lrs4Δ::KanMX4*	This study	From DMY2804
	SY610	*MATa ura3-1 leu2-3,112 trp1-1 his3-11,15 ade2-1 can1-100 RDN1-NTS2::mURA3 lrs4Δ::KanMX4*	This study	Form DMY2800
	SY636[b]	*MATa ura3-1 leu2-3,112 trp1-1 his3-11,15 ade2-1 can1-100 leu2::mURA3 lrs4Δ::KanMX4 jhd2Δ::HIS3MX6*	This study	From DMY2798
	SY642[b]	*MATa ura3-1 leu2-3,112 trp1-1 his3-11,15 ade2-1 can1-100 RDN1-NTS1::mURA3 lrs4Δ::KanMX4 jhd2Δ::HIS3MX6*	This study	From DMY2804
	SY648[b]	*MATa ura3-1 leu2-3,112 trp1-1 his3-11,15 ade2-1 can1-100 RDN1-NTS2::mURA3 lrs4Δ::KanMX4 jhd2Δ::HIS3MX6*	This study	Form DMY2800
Figure 5A	W303R (W303AR5)	*MATa ura3-1 leu2-3,112 his3-11,15 trp1-1 ade2-1 can1-100 RAD5$^+$ RDN1::ADE2*	Won-Ki Huh & Leonard Guarente	[8,9]
	SY562	*MATa ura3-1 leu2-3,112 his3-11,15 trp1-1 ade2-1 can1-100 RAD5$^+$ RDN1::ADE2 set1Δ::KanMX4*	This study	From W303R
	SY563	*MATa ura3-1 leu2-3,112 his3-11,15 trp1-1 ade2-1 can1-100 RAD5$^+$ RDN1::ADE2 set2Δ::KanMX4*	This study	From W303R
	SY564	*MATa ura3-1 leu2-3,112 his3-11,15 trp1-1 ade2-1 can1-100 RAD5$^+$ RDN1::ADE2 dot1Δ::KanMX4*	This study	From W303R
	SY549	*MATa ura3-1 leu2-3,112 his3-11,15 trp1-1 ade2-1 can1-100 RAD5$^+$ RDN1::ADE2 jhd2Δ::KanMX4*	This study	From W303R

	SY550	*MATa ura3-1 leu2-3,112 his3-11,15 trp1-1 ade2-1 can1-100 RAD5$^+$ RDN1::ADE2 gis1Δ::KanMX4*	This study	From W303R
	SY551	*MATa ura3-1 leu2-3,112 his3-11,15 trp1-1 ade2-1 can1-100 RAD5$^+$ RDN1::ADE2 sir2Δ::KanMX4*	This study	From W303R
	SY552	*MATa ura3-1 leu2-3,112 his3-11,15 trp1-1 ade2-1 can1-100 RAD5$^+$ RDN1::ADE2 fob1Δ::KanMX4*	This study	From W303R
	SY553	*MATa ura3-1 leu2-3,112 his3-11,15 trp1-1 ade2-1 can1-100 RAD5$^+$ RDN1::ADE2 tof2Δ::KanMX4*	This study	From W303R
	SY554	*MATa ura3-1 leu2-3,112 his3-11,15 trp1-1 ade2-1 can1-100 RAD5$^+$ RDN1::ADE2 csm1Δ::KanMX4*	This study	From W303R
	SY555	*MATa ura3-1 leu2-3,112 his3-11,15 trp1-1 ade2-1 can1-100 RAD5$^+$ RDN1::ADE2 lrs4Δ::KanMX4*	This study	From W303R
Figure 5C	W303R	See Figure 5A		
	SY549	See Figure 5A		
	SY551	See Figure 5A		
	SY570[b]	*MATa ura3-1 leu2-3,112 his3-11,15 trp1-1 ade2-1 can1-100 RAD5$^+$ RDN1::ADE2 sir2Δ::KanMX4 jhd2Δ::HIS3MX6*	This study	From W303R
	SY553	See Figure 5A		
	SY572[b]	*MATa ura3-1 leu2-3,112 his3-11,15 trp1-1 ade2-1 can1-100 RAD5$^+$ RDN1::ADE2 tof2Δ::KanMX4 jhd2Δ::HIS3MX6*	This study	From W303R
Figure 6A	FY416	*MATa ura3Δ0 leu2Δ0 his3Δ1 met15Δ0 FOB1-TAP tag::HIS3MX6*	Euroscarf	
	SY517	*MATa ura3Δ0 leu2Δ0 his3Δ1 met15Δ0 jhd2Δ::KanMX4 FOB1-TAP tag::HIS3MX6*	This study	
	SY521	*MATa his3Δ1 leu2Δ0 met15Δ0 ura3Δ0 gis1Δ::KanMX4 FOB1-TAP tag::HIS3MX6*	This study	
Figure 6B	FY417	*MATa ura3Δ0 leu2Δ0 his3Δ1 met15Δ0 TOF2-TAP tag::HIS3MX6*	Euroscarf	
	SY518	*MATa ura3Δ0 leu2Δ0 his3Δ1 met15Δ0 jhd2Δ::KanMX4 TOF2-TAP tag::HIS3MX6*	This study	
	SY522	*MATa ura3Δ0 leu2Δ0 his3Δ1 met15Δ0 gis1Δ::KanMX4 TOF2-TAP tag::HIS3MX6*	This study	
Figure 6C	FY418	*MATa ura3Δ0 leu2Δ0 his3Δ1 met15Δ0 CSM1-TAP tag::HIS3MX6*	Euroscarf	
	SY519	*MATa ura3Δ0 leu2Δ0 his3Δ1 met15Δ0 jhd2Δ::KanMX4 CSM1-TAP tag::HIS3MX6*	This study	
	SY523	*MATa ura3Δ0 leu2Δ0 his3Δ1 met15Δ0 gis1Δ::KanMX4 CSM1-TAP tag::HIS3MX6*	This study	
Figure 6D	FY419	*MATa ura3Δ0 leu2Δ0 his3Δ1 met15Δ0 LRS4-TAP tag::HIS3MX6*	Euroscarf	
	SY520	*MATa ura3Δ0 leu2Δ0 his3Δ1 met15Δ0 jhd2Δ::KanMX4 LRS4-TAP tag::HIS3MX6*	This study	
	SY524	*MATa ura3Δ0 leu2Δ0 his3Δ1 met15Δ0 gis1Δ::KanMX4 LRS4-TAP tag::HIS3MX6*	This study	
Figure 6E	FY452	*MATa ura3Δ0 leu2Δ0 his3Δ1 met15Δ0 SMC4-TAP tag::HIS3MX6*	Euroscarf	
	SY625	*MATa ura3Δ0 leu2Δ0 his3Δ1 met15Δ0 jhd2Δ::KanMX4 SMC4-TAP tag::HIS3MX6*	This study	
	SY628	*MATa ura3Δ0 leu2Δ0 his3Δ1 met15Δ0 gis1Δ::KanMX4 SMC4-TAP tag::HIS3MX6*	This study	
Figure 6F	FY454	*MATa ura3Δ0 leu2Δ0 his3Δ1 met15Δ0 BRN1-TAP tag::HIS3MX6*	Euroscarf	
	SY627	*MATa ura3Δ0 leu2Δ0 his3Δ1 met15Δ0 jhd2Δ::KanMX4 BRN1-TAP tag::HIS3MX6*	This study	
	SY630	*MATa ura3Δ0 leu2Δ0 his3Δ1 met15Δ0 gis1Δ::KanMX4 BRN1-TAP tag::HIS3MX6*	This study	
Figure 7A	BY4741	*MATa ura3Δ0 leu2Δ0 his3Δ1 met15Δ0*		
	FY187	*MATa ura3Δ0 leu2Δ0 his3Δ1 met15Δ0 jhd2Δ::KanMX4*	Open Biosystems	
	FY189	*MATa ura3Δ0 leu2Δ0 his3Δ1 met15Δ0 gis1Δ::KanMX4*	Open Biosystems	
Figure 7C	SY702	See Figure 1H and 1I		
	SY703	See Figure 1H and 1I		
	SY704	See Figure 1H and 1I		

Figure 7E	FY187	See Figure 7A		
	SY703	See Figure 1H and 1I		
Figure S1	UCC7262	*MATa ura3(Δ0 or -52) leu2(Δ0 or Δ1) trp1Δ63 his3Δ200 ade2(::hisG or -101) met15Δ0 or MET15 lys2(Δ0 or -801) ADE2-TEL-VR hmr::URA3 hhf2-hht2::MET15 hhf1-hht1::LEU2* pMP9 (*LYS2 CEN ARS*) - *HHF2-HHT2*	Daniel E. Gottschling	[6]
	SY453	*MATa ura3(Δ0 or -52) leu2(Δ0 or Δ1) trp1Δ63 his3Δ200 ade2(::hisG or -101) met15Δ0 or MET15 lys2(Δ0 or -801) ADE2-TEL-VR hmr::URA3 hhf2-hht2::MET15 hhf1-hht1::LEU2 jhd1Δ::KanMX4* pMP9 (*LYS2 CEN ARS*) - *HHF2-HHT2*	This study	From UCC7262
	SY383	*MATa ura3(Δ0 or -52) leu2(Δ0 or Δ1) trp1Δ63 his3Δ200 ade2(::hisG or -101) met15Δ0 or MET15 lys2(Δ0 or -801) ADE2-TEL-VR hmr::URA3 hhf2-hht2::MET15 hhf1-hht1::LEU2 jhd2Δ::KanMX4* pMP9 (*LYS2 CEN ARS*) - *HHF2-HHT2*	This study	From UCC7262
	SY454	*MATa ura3(Δ0 or -52) leu2(Δ0 or Δ1) trp1Δ63 his3Δ200 ade2(::hisG or -101) met15Δ0 or MET15 lys2(Δ0 or -801) ADE2-TEL-VR hmr::URA3 hhf2-hht2::MET15 hhf1-hht1::LEU2 rph1Δ::KanMX4* pMP9 (*LYS2 CEN ARS*) - *HHF2-HHT2*	This study	From UCC7262
	SY455	*MATa ura3(Δ0 or -52) leu2(Δ0 or Δ1) trp1Δ63 his3Δ200 ade2(::hisG or -101) met15Δ0 or MET15 lys2(Δ0 or -801) ADE2-TEL-VR hmr::URA3 hhf2-hht2::MET15 hhf1-hht1::LEU2 ecm5Δ::KanMX4* pMP9 (*LYS2 CEN ARS*) - *HHF2-HHT2*	This study	From UCC7262
	SY456	*MATa ura3(Δ0 or -52) leu2(Δ0 or Δ1) trp1Δ63 his3Δ200 ade2(::hisG or -101) met15Δ0 or MET15 lys2(Δ0 or -801) ADE2-TEL-VR hmr::URA3 hhf2-hht2::MET15 hhf1-hht1::LEU2 gis1Δ::KanMX4* pMP9 (*LYS2 CEN ARS*) - *HHF2-HHT2*	This study	From UCC7262
	SY346	*MATa ura3(Δ0 or -52) leu2(Δ0 or Δ1) trp1Δ63 his3Δ200 ade2(::hisG or -101) met15Δ0 or MET15 lys2(Δ0 or -801) ADE2-TEL-VR hmr::URA3 hhf2-hht2::MET15 hhf1-hht1::LEU2 sir2Δ::KanMX4* pMP9 (*LYS2 CEN ARS*) - *HHF2-HHT2*	[5]	From UCC7262
	SY369	*MATa ura3(Δ0 or -52) leu2(Δ0 or Δ1) trp1Δ63 his3Δ200 ade2(::hisG or -101) met15Δ0 or MET15 lys2(Δ0 or -801) ADE2-TEL-VR hmr::URA3 hhf2-hht2::MET15 hhf1-hht1::LEU2 rpd3Δ::KanMX6* pMP9 (*LYS2 CEN ARS*) - *HHF2-HHT2*	[5]	From UCC7262
	UCC7266	*MATa ura3(Δ0 or -52) leu2(Δ0 or Δ1) trp1Δ63 his3Δ200 lys2(Δ0 or -801) ADE2-TEL-VR hml::URA3 hhf2-hht2::MET15 hhf1-hht1::LEU2* pMP9 (*LYS2 CEN ARS*) - *HHF2-HHT2*	Daniel E. Gottschling	[6]
	SY465	*MATa ura3(Δ0 or -52) leu2(Δ0 or Δ1) trp1Δ63 his3Δ200 lys2(Δ0 or -801) ADE2-TEL-VR hml::URA3 hhf2-hht2::MET15 hhf1-hht1::LEU2 jhd1Δ::KanMX4* pMP9 (*LYS2 CEN ARS*) - *HHF2-HHT2*	This study	From UCC7262
	SY384	*MATa ura3(Δ0 or -52) leu2(Δ0 or Δ1) trp1Δ63 his3Δ200 lys2(Δ0 or -801) ADE2-TEL-VR hml::URA3 hhf2-hht2::MET15 hhf1-hht1::LEU2 jhd2Δ::KanMX4* pMP9 (*LYS2 CEN ARS*) - *HHF2-HHT2*	This study	From UCC7262
	SY466	*MATa ura3(Δ0 or -52) leu2(Δ0 or Δ1) trp1Δ63 his3Δ200 lys2(Δ0 or -801) ADE2-TEL-VR hml::URA3 hhf2-hht2::MET15 hhf1-hht1::LEU2 rph1Δ::KanMX4* pMP9 (*LYS2 CEN ARS*) - *HHF2-HHT2*	This study	From UCC7262
	SY467	*MATa ura3(Δ0 or -52) leu2(Δ0 or Δ1) trp1Δ63 his3Δ200 lys2(Δ0 or -801) ADE2-TEL-VR hml::URA3 hhf2-hht2::MET15 hhf1-hht1::LEU2 ecm5Δ::KanMX4* pMP9 (*LYS2 CEN ARS*) - *HHF2-HHT2*	This study	From UCC7262
	SY468	*MATa ura3(Δ0 or -52) leu2(Δ0 or Δ1) trp1Δ63 his3Δ200 lys2(Δ0 or -801) ADE2-TEL-VR hml::URA3 hhf2-hht2::MET15 hhf1-hht1::LEU2 gis1Δ::KanMX4* pMP9 (*LYS2 CEN ARS*) - *HHF2-HHT2*	This study	From UCC7262
	SY347	*MATa ura3(Δ0 or -52) leu2(Δ0 or Δ1) trp1Δ63 his3Δ200 lys2(Δ0 or -801) ADE2-TEL-VR hml::URA3 hhf2-*	[5]	From UCC7262

		hht2::MET15 hhf1-hht1::LEU2 sir2Δ::KanMX4 pMP9 (*LYS2 CEN ARS*) - *HHF2-HHT2*		
	SY370	*MATa ura3(Δ0 or -52) leu2(Δ0 or Δ1) trp1Δ63 his3Δ200 lys2(Δ0 or -801) ADE2-TEL-VR hml::URA3 hhf2-hht2::MET15 hhf1-hht1::LEU2 rpd3Δ::KanMX6* pMP9 (*LYS2 CEN ARS*) - *HHF2-HHT2*	[5]	From UCC7262
Figure S2	FY366	See Figure 2B		
	SY482	See Figure 2B		
	SY483	See Figure 2B		
	SY484	See Figure 2B		
	SY485	See Figure 2B		
	SY486	See Figure 2B		
	SY487	See Figure 2B		
	SY488	See Figure 2B		
	SY489	See Figure 2B		
	SY490	See Figure 2B		
Figure S3	SY565	See Figure 3C		
	SY566	See Figure 3C		
	SY567	See Figure 3C		
Figure S4	W303R	See Figure 5A		
	SY549 (*jhd2Δ* #1)	See Figure 5A		
	SY569[b] (*jhd2Δ* #2)	*MATa ura3-1 leu2-3,112 his3-11,15 trp1-1 ade2-1 can1-100 $RAD5^+$ RDN1::ADE2 jhd2Δ::HIS3MX6*	This study	From W303R
	SY570	See Figure 5C		
	SY571[b]	*MATa ura3-1 leu2-3,112 his3-11,15 trp1-1 ade2-1 can1-100 $RAD5^+$ RDN1::ADE2 fob1Δ::KanMX4 jhd2Δ::HIS3MX6*	This study	From W303R
	SY572	See Figure 5C		
	SY573[b]	*MATa ura3-1 leu2-3,112 his3-11,15 trp1-1 ade2-1 can1-100 $RAD5^+$ RDN1::ADE2 csm1Δ::KanMX4 jhd2Δ::HIS3MX6*	This study	From W303R
	SY574[b]	*MATa ura3-1 leu2-3,112 his3-11,15 trp1-1 ade2-1 can1-100 $RAD5^+$ RDN1::ADE2 lrs4Δ::KanMX4 jhd2Δ::HIS3MX6*	This study	From W303R

[a] YJR119C is the systematic name for *JHD2*.

[b] The *JHD2* deletion cassette was amplified from strain SY568 (*MATa ura3Δ0 leu2Δ0 his3Δ1 met15Δ0 jhd2*Δ*::HIS3MX6*).

Table S2. Plasmids used in this study.

Name	Description	Source & Reference
pFA6a-His3MX6	*S. kluyveri HIS3*	Mark S. Longtine [10]
pRS424	*2μ*, *TRP1*	ATCC
pRS424-YJR119c-3XHA[a]	*2μ*, *TRP1, YJR119c-HA*$_3$	TaeSoo Kim [11]
pRS424-yjr119c-3XHA(H427A)[a]	*2μ*, *TRP1, yjr119c(H427A)-HA*$_3$	TaeSoo Kim
pWZ414-F13	*CEN*, *TRP1*, *HHT2-HHF2*	Eun-Jung Cho & Sharon Y.Roth [12,13]
pRS314-H3(K4A)-H4	*CEN*, *TRP1*, *hht2-K4A-HHF2*	Eun-Jung Cho & Stephen Buratowski [13]
pRS314-H3(K36A)-H4	*CEN*, *TRP1*, *hht2-K36A-HHF2*	Eun-Jung Cho & Stephen Buratowski [13]
pWZ414-F13-H3(K79A)-H4	*CEN*, *TRP1*, *hht2-K79A-HHF2*	Eun-Jung Cho [13]
pRS325-GALpro	*2μ*, *LEU2*, P_{GAL10}	TaeSoo Kim & Dave Miller/Gerry Fink [11]
pRS325-GALpro-YJR119c-3XHA[a]	*2μ*, *LEU2*, P_{GAL10}-*YJR119c-HA*$_3$	TaeSoo Kim [11]
pRS325-GALpro-GIS1-3XHA	*2μ*, *LEU2*, P_{GAL10}-*GIS1-HA*$_3$	TaeSoo Kim [11]
p362	*Bgl*II fragment containing the 5' half of an rDNA unit	Frank Uhlmann & G. Shirleen Roeder [14,15]

[a] YJR119C is the systematic name for *JHD2*.

Table S3 Oligonucleotide sequences used in ChIP analysis.

Name	Sequence	Reference
TEL07L-URA3-1-for (123- 144)[a]	CCCAGCCTGCTTTTCTGTAACG	[5]
TEL07L-URA3-1-rev (306-329)[a]	TGGGTGGAAGAGATGAAGGTTACG	[5]
TEL07L-URA3-2-for (732- 756)[a]	TGGGACCTAATGCTTCAACTAACTC	[5]
TEL07L-URA3-2-rev (1003-1024)[a]	GGAAGAACGAAGGAAGGAGCAC	[5]
TEL07L-URA3-3-for (4912- 4933)[a]	ATTTCCTCTCCCTGCCATCCTC	[5]
TEL07L-URA3-3-rev (5141-5164)[a]	GCTCTCCTCCCACAAAATAATCTT	[5]
TEL07L-URA3-4-for (15987-16009)[a]	GAATAATCGGCTGTAATCGGACC	[5]
TEL07L-URA3-4-rev (16261-16281)[a]	ACCAACCGGATCAGGCAAGAC	[5]
rDNA-2-for (ChXII 460408-460427)	TCCCCACTGTTCACTGTTCA	[7]
rDNA-2-rev (ChXII 460632-460651)	AGGGCTTTCACAAAGCTTCC	[7]
rDNA-4-for (ChXII 458177-458196)	AAGATGCCCACGATGAGACT	[7]
rDNA-4-rev (ChXII 458425-458444)	GGGAGGTACTTCATGCGAAA	[7]
ChrV NO-ORF-for[b]	GGCTGTCAGAATATGGGGCCGTAGTA	[16,17]
ChrV NO-ORF-rev[b]	CACCCCGAAGCTGCTTTCACAATAC	[16,17]

[a] The nucleotide numbers are relative to the first nucleotide of the initiation codon (+1) of *URA3* integrated at TEL07L.

[b] The primers used to amplify the non-transcribed regions of chromosome V as an internal control.

Table S4 List of antibodies used in this study.

Specificity	Supplier	Catalog #	Application
Anti-Sir2	Santa Cruz Biotechnology	sc-6666	ChIP
Anti-Rap1	Santa Cruz Biotechnology	sc-20167	ChIP
Anti-H3K36me3	Abcam	ab9050	ChIP
Anti-H3K36me2	Millipore	07-369	ChIP
Anti-H3K36me1	Abcam	ab9048	ChIP
Anti-H3K4me3	Abcam	ab8580	ChIP, WB
Anti-H3K4me2	Abcam	ab7766	ChIP
Anti-H3K4me1	Abcam	ab8895	ChIP
Anti-H3	Abcam	ab1791	ChIP, WB
Anti-HA	Roche	11583816001	WB
Anti-digoxigenin	Roche	11333062910	FISH
FITC–anti-mouse	Jackson Immunoresearch	115-095-003	FISH
FITC–anti-goat	Jackson Immunoresearch	705-095-003	FISH

Supplementary references

1. van Welsem T, Frederiks F, Verzijlbergen KF, Faber AW, Nelson ZW, Egan DA, Gottschling DE, van Leeuwen F: **Synthetic lethal screens identify gene silencing processes in yeast and implicate the acetylated amino terminus of Sir3 in recognition of the nucleosome core**. *Mol Cell Biol* 2008, **28**:3861-3872.
2. Kahana A, Gottschling DE: **DOT4 links silencing and cell growth in Saccharomyces cerevisiae**. *Mol Cell Biol* 1999, **19**:6608-6620.
3. Ng HH, Feng Q, Wang HB, Erdjument-Bromage H, Tempst P, Zhang Y, Struhl K: **Lysine methylation within the globular domain of histone H3 by Dot1 is important for telomeric silencing and Sir protein association**. *Genes & development* 2002, **16**:1518-1527.
4. Kelly TJ, Qin S, Gottschling DE, Parthun MR: **Type B histone acetyltransferase Hat1p participates in telomeric silencing**. *Mol Cell Biol* 2000, **20**:7051-7058.
5. Rhie BH, Song YH, Ryu HY, Ahn SH: **Cellular aging is associated with increased ubiquitylation of histone H2B in yeast telomeric heterochromatin**. *Biochem Biophys Res Commun* 2013, **439**:570-575.
6. van Leeuwen F, Gafken PR, Gottschling DE: **Dot1p modulates silencing in yeast by methylation of the nucleosome core**. *Cell* 2002, **109**:745-756.
7. Huang J, Brito IL, Villen J, Gygi SP, Amon A, Moazed D: **Inhibition of homologous recombination by a cohesin-associated clamp complex recruited to the rDNA recombination enhancer**. *Genes & development* 2006, **20**:2887-2901.
8. Ha CW, Sung MK, Huh WK: **Nsi1 plays a significant role in the silencing of ribosomal DNA in Saccharomyces cerevisiae**. *Nucleic acids research* 2012, **40**:4892-4903.
9. Mills KD, Sinclair DA, Guarente L: **MEC1-dependent redistribution of the Sir3 silencing protein from telomeres to DNA double-strand breaks**. *Cell* 1999, **97**:609-620.
10. Longtine MS, McKenzie A, Demarini DJ, Shah NG, Wach A, Brachat A, Philippsen P, Pringle JR: **Additional modules for versatile and economical PCR-based gene deletion and modification in Saccharomyces cerevisiae**. *Yeast* 1998, **14**:953-961.
11. Kim T, Buratowski S: **Two Saccharomyces cerevisiae JmjC domain proteins demethylate histone H3 Lys36 in transcribed regions to promote elongation**. *Journal of Biological Chemistry* 2007, **282**:20827-20835.
12. Zhang WZ, Bone JR, Edmondson DG, Turner BM, Roth SY: **Essential and redundant functions of histone acetylation revealed by mutation of target lysines and loss of the Gcn5p acetyltransferase**. *Embo J* 1998, **17**:3155-3167.
13. Seol JH, Kim HJ, Yang YJ, Kim ST, Youn HD, Han JW, Lee HW, Cho EJ: **Different roles of histone H3 lysine 4 methylation in chromatin maintenance**. *Biochem Biophys Res Commun* 2006, **349**:463-470.
14. Keil RL, Roeder GS: **Cis-Acting, Recombination-Stimulating Activity in a Fragment of the Ribosomal DNA of S-Cerevisiae**. *Cell* 1984, **39**:377-386.
15. Sullivan M, Higuchi T, Katis VL, Uhlmann F: **Cdc14 phosphatase induces rDNA condensation and resolves cohesin-independent cohesion during budding yeast anaphase**. *Cell* 2004, **117**:471-482.
16. Ahn SH, Kim M, Buratowski S: **Phosphorylation of serine 2 within the RNA polymerase IIC-terminal domain couples transcription and 3 ' end processing**. *Mol Cell* 2004, **13**:67-76.

17. Song YH, Ahn SH: **A Bre1-associated Protein, Large 1 (Lge1), Promotes H2B Ubiquitylation during the Early Stages of Transcription Elongation**. *J Biol Chem* 2010, **285**:2361-2367.
